# A new *Mourasuchus* (Alligatoroidea, Caimaninae) from the late Miocene of Venezuela, the phylogeny of Caimaninae and considerations on the feeding habits of *Mourasuchus*

**DOI:** 10.7717/peerj.3056

**Published:** 2017-03-07

**Authors:** Giovanne M. Cidade, Andrés Solórzano, Ascanio Daniel Rincón, Douglas Riff, Annie Schmaltz Hsiou

**Affiliations:** 1 Departamento de Biologia, FFCLRP, Universidade de São Paulo, Ribeirão Preto, São Paulo, Brazil; 2 Centro de Ecología, Instituto Venezolano de Investigaciones Científcas, San António de los Altos, Miranda, Venezuela; 3 Instituto de Biologia, Universidade Federal de Uberlândia, Uberlândia, Minas Gerais, Brazil

**Keywords:** *Mourasuchus*, Caimaninae, Alligatoroidea, Phylogeny, *Mourasuchus pattersoni*, Feeding behaviour, Miocene, South America, Paleoecology

## Abstract

*Mourasuchus* (Alligatoroidea, Caimaninae) is one of the most peculiar crocodyliforms due to the skull morphology consisting of a long, wide, dorsoventrally flat rostrum with long, slender mandibular rami. Despite these peculiarities, the systematics, phylogeny and feeding habits of this taxon have not been properly studied. In this paper, we describe a new species of the genus, *Mourasuchus pattersoni* sp. nov., from the late Miocene of the Urumaco Formation of Venezuela. The new species differs from the other *Mourasuchus* species in having a lateromedially wide, dorsoventrally high jugal bone and a circular incisive foramen, which both represent autapomorphies of the new taxon. Phylogenetically, *M. pattersoni* sp. nov. is more closely related to *M. amazonensis* and the specimen UFAC-1424 (formely attributed to *M. nativus*) than to *M. arendsi* or *M. atopus*, whilst *Mourasuchus* is recovered once more as a monophyletic group. Furthermore, the cladistic analysis performed in this contribution offers a new phylogenetic assessment of Caimaninae, including many taxa described recently for the group. In this study, we also discuss the crocodylian diversity of the Urumaco Formation as well as how paleoenvironment may have contributed toward its evolution. In addition, we provide a discussion of the potential feeding habits of *Mourasuchus*. In this contribution, *Mourasuchus* is regarded as a taxon that likely preferred to prey on small animals. The unusual skull morphology of this group may have evolved to cover a large area with the rostrum, allowing for a more efficient prey capture, while the prey may have consisted predominantly of large amounts of small animals.

## Introduction

Alligatoroidea represents a clade formed by *Alligator mississippiensis* (Daudin, 1802) and all crocodylians closer to it than to *Gavialis gangeticus* (Gmelin, 1789) and *Crocodylus niloticus* Laurenti, 1768 (Brochu, 2003). Alligatoroidea itself is divided into two main clades: Alligatorinae, formed by the taxa closer to *Alligator mississippiensis* than to *Caiman crocodilus* (Linnaeus, 1758); and Caimaninae, formed by the taxa closer to *Caiman crocodilus* than to *Alligator mississippiensis* (Brochu, 2003). Caimaninae currently comprises six species distributed among the genera *Caiman*—*C. crocodilus*, *C. yacare* (Daudin, 1802) and *C. latirostris* (Daudin, 1802)—, *Paleosuchus*—*P. trigonatus* (Schneider, 1801) and *P. palpebrosus* (Cuvier, 1807) and *Melanosuchus*—*M. niger* (Spix, 1825). However, the fossil record of the group shows a much richer diversity (e.g., Langston, 1965; Brochu, 1999; Brochu, 2010; Brochu, 2011; Riff et al., 2010; Bona, Riff & Gasparini, 2013; Bona & Barrios, 2015), which traces back unequivocally to the early Paleocene of Argentina (Bona, 2007; Brochu, 2011). While potential caimanine remains have been reported from the Late Cretaceous and the Paleocene of North America (Bryant, 1989; Brochu, 1996), their relationship to other alligatoroids remains untested (Brochu, 2010).

As with the extant species, the fossil diversity of Caimaninae is predominantly South American. Notable exceptions of this are *Orthogenysuchus olseni* Mook, 1924 and *Tsoabichi greenriverensis* Brochu, 2010 from the Eocene of the United States (Brochu, 2010), as well as *Culebrasuchus mesoamericanus* Hastings et al., 2013 and *Centenariosuchus gilmorei* Hastings et al., 2013, from the early Miocene of Panama (Hastings et al., 2013; Hastings, Reisser & Scheyer, 2016). The phylogeny of Caimaninae has been extensively evaluated in the past few years (Brochu, 1999; Brochu, 2010; Brochu, 2011; Aguilera, Riff & Bocquentin-Villanueva, 2006; Bona, 2007; Bona, Riff & Gasparini, 2013) although comprehensive analyses involving some recently described forms (e.g.,  Pinheiro et al., 2012; Bona & Carabajal, 2013; Hastings et al., 2013; Scheyer et al., 2013; Fortier et al., 2014; Salas-Gismondi et al., 2015) are still lacking (e.g., Salas-Gismondi et al., 2015; Hastings, Reisser & Scheyer, 2016).

The fossil record shows the Miocene as the time when Caimaninae reached the apex of both its diversity and its morphological disparity. The units that yield the richest and most diverse fossil records of the group are the deposits of the Honda Group in Colombia (Langston, 1965; Langston & Gasparini, 1997) and of the Pebas Formation in Peru (Salas-Gismondi et al., 2015) in the middle Miocene, and those of the Ituzaingó Formation in Argentina (Bona, Riff & Gasparini, 2013), the Urumaco Formation in Venezuela (Aguilera, 2004; Riff et al., 2010) and the Solimões Formation in Brazil (Riff et al., 2010) in the late Miocene. The South American Miocene caimanine fossil record contains, among others, two highly distinctive, peculiar forms. One of them is the giant, apex predator *Purussaurus* (Barbosa-Rodrigues, 1892; Mook, 1941; Langston, 1965; Price, 1967; Bocquentin-Villanueva et al., 1989; Aguilera, Riff & Bocquentin-Villanueva, 2006). The other is the “duck-faced” genus *Mourasuchus*, which traditionally included four species restricted to the Miocene of South America: *M. atopus* (Langston, 1965), from the middle Miocene of Colombia and Peru (Langston, 1965; Langston & Gasparini, 1997; Salas-Gismondi et al., 2007; Salas-Gismondi et al., 2015); *M. amazonensis* Price, 1964, from the late Miocene of Brazil (Price, 1964; Souza-Filho & Guilherme, 2011a); *M. nativus* (Gasparini, 1985), from the late Miocene of Argentina, Brazil and Venezuela (Gasparini, 1985; Bocquentin & Souza-Filho, 1990; Bona, Degrange & Fernández, 2013; Cidade et al., 2013; Scheyer et al., 2013); and *M. arendsi* Bocquentin-Villanueva, 1984 from the late Miocene of Venezuela and Brazil (Bocquentin-Villanueva, 1984; Souza-Filho & Guilherme, 2011b). However, Scheyer & Delfino (2016) considered *M. nativus* as a junior synonym of *M. arendsi*, bringing the number of currently known *Mourasuchus* species to three. This genus has a peculiar morphology, especially in the skull, which exhibits a long, wide, dorsoventrally flat rostrum (which may be classified as “platyrostral-broad” following Busbey, 1994) together with a relatively small skull table (Bona, Riff & Gasparini, 2013). The mandibles of *Mourasuchus* are also long and markedly slender, with a short mandibular symphysis that only extends to the level of the first alveolous. Some postcranial features of *Mourasuchus* may also be considered unusual, such as cervical vertebrae relatively shorter than in extant crocodylians (Bocquentin-Villanueva, 1984) with small, non-hooked hypapophyses and low neural spines (Langston, 2008). These anatomical features, in particular those of the cranium and mandible, indicate that *Mourasuchus* was not able to capture, hold and dismember large prey, like most extant crocodylians (Langston, 1965; Langston, 2008). Because of this inability to process food in typical crocodylian-fashion, previous researchers have suggested that *Mourasuchus* may have been a “filter feeder” (Langston, 1965; Riff et al., 2010; Bona, Degrange & Fernández, 2013), although a detailed method of how this “filtering” was performed has not yet been described.

This paper describes a new species, *Mourasuchus pattersoni* sp. nov., from the late Miocene of the Urumaco Formation of Venezuela. The holotype, MCNC-PAL-110-72V, is a nearly complete skull with both mandibular rami, and several postcranial remains (atlas, axis, two sacral and the first caudal vertebrae along with tentatively identified seven cervical, six thoracic, three lumbar and 13 caudal vertebrae, five cervical and an unknown number of sacral and caudal ribs along with fragments of thoracic ribs, both scapulae and coracoids, a right ilium and ischium and 15 osteoderms). The postcranial remains of MCNC-PAL-110-72V were described by Langston (2008), but the skull and mandibles were not available to him at the time. As a result, he stated the specimen “probably” belonged to *Mourasuchus arendsi*. However, the morphological study of the cranium and mandibles of MCNC-PAL-110-72V performed in this contribution allowed us to conclude that this specimen represents a new species of *Mourasuchus*. Additionally, this paper also provides a comprehensive phylogenetic analysis of Caimaninae, including many of the most recently described fossil taxa, as well as comments on the paleoecology and feeding habits of *Mourasuchus*.

## Materials and Methods

**Figure 1:**
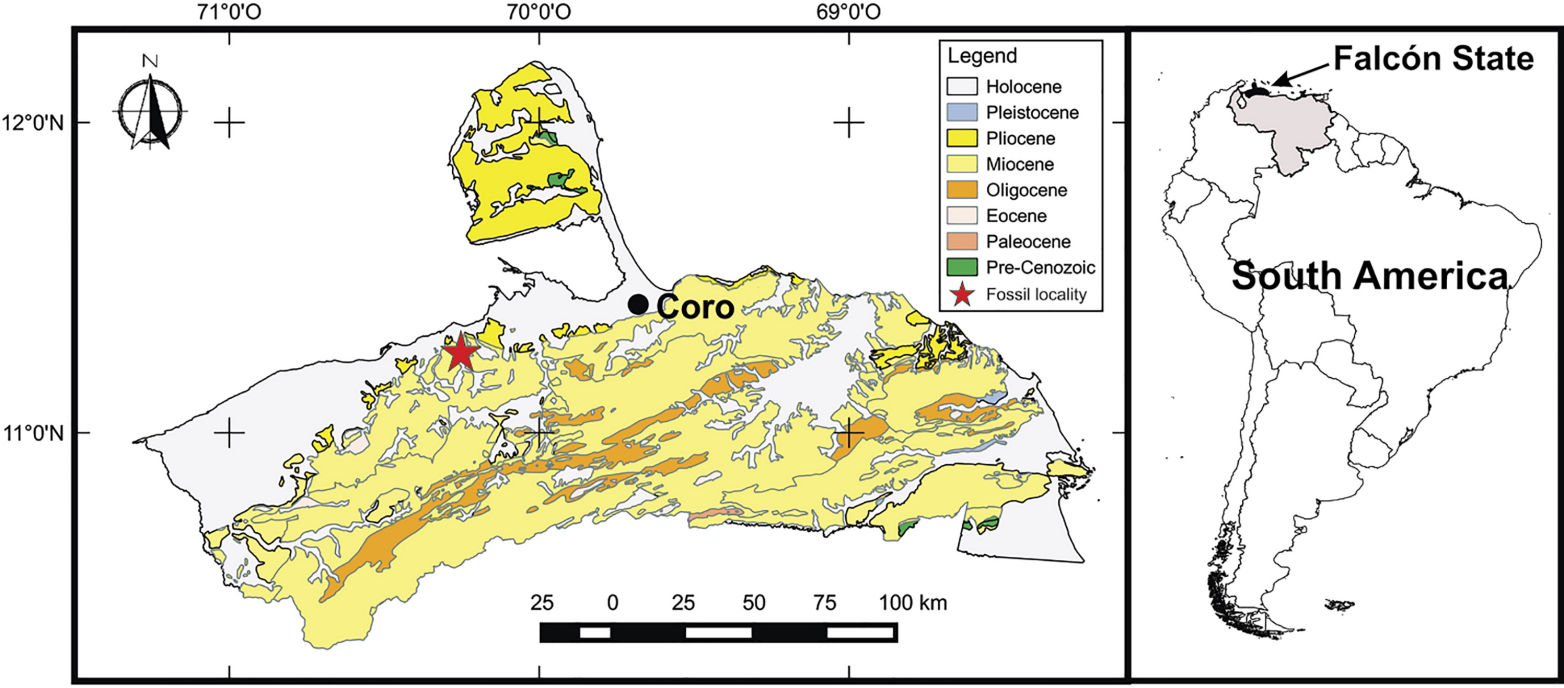
Map of the fossil locality in Venezuela. Map of the Venezuelan state of Falcón showing the fossil locality within the Miocene deposits. From Cáceres et al. (2016).

The holotype of *Mourasuchus pattersoni* sp. nov. (MCNC-PAL-110-72V) was collected in the late Miocene deposits of the Urumaco Formation, Venezuela, in 1972 by a joint expedition of American and Venezuelan institutions led by eminent paleontologist Dr. Bryan Patterson (see Linares, 2004). There is a discrepancy (see Langston, 2008) about whether the specimen was collected in deposits of the Lower or of the Upper Members of the Urumaco Formation. Until further information clarifies this issue, we follow Langston (2008) in considering that MCNC-PAL-110-72V was recovered in the stratum better known informally as “*capa de huesos*” (“layer of bones”) or “*capa de tortugas*” (“layer of turtles”), located in the Upper Member of the Urumaco Formation (Royo y Gómez, 1960; Linares, 2004; Fig. 1). This interpretation concurs better with the information given in Patterson’s field notes about the locality where the specimen was collected (see Langston, 2008, p. 126).

Both the cranial and postcranial remains of MCNC-PAL-110-72V were sent to the Museum of Comparative Zoology (MCZ) at Harvard University, where the skull and mandibles underwent restoration that covered several areas of the bone with plaster (including the addition of artificial teeth, also made of plaster, in both maxillae and both mandibular rami). The addition of plaster has obscured parts of the specimen, yet this did not prevent a comprehensive anatomical, taxonomic, and phylogenetic study to be performed.

**Figure 2:**
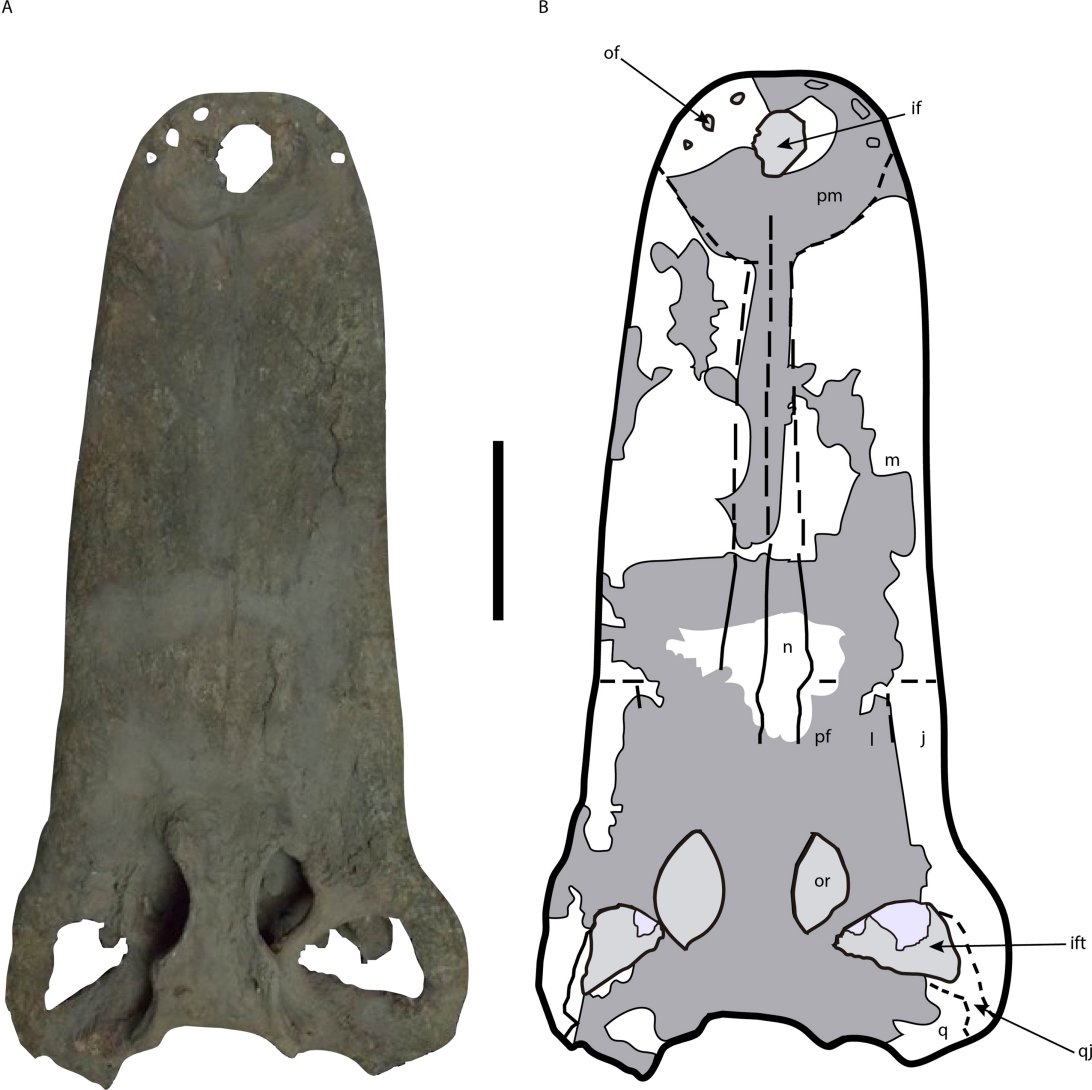
Skull of *Mourasuchus pattersoni* sp. nov. (MCNC-PAL-110-72V, holotype) in dorsal view (A) with schematic drawing (B). In dark grey, the areas covered by plaster. Abbreviations: if, incisive foramen; ift, infratemporal fenestra; j, jugal; l, lacrimal; m, maxilla; n, nasal; of, occlusal fossa; or, orbit; pf, prefrontal; pm, premaxilla; pt: pterygoid; q, quadrate; qj, quadratojugal. Scale bar equals 20 cm.

**Figure 3:**
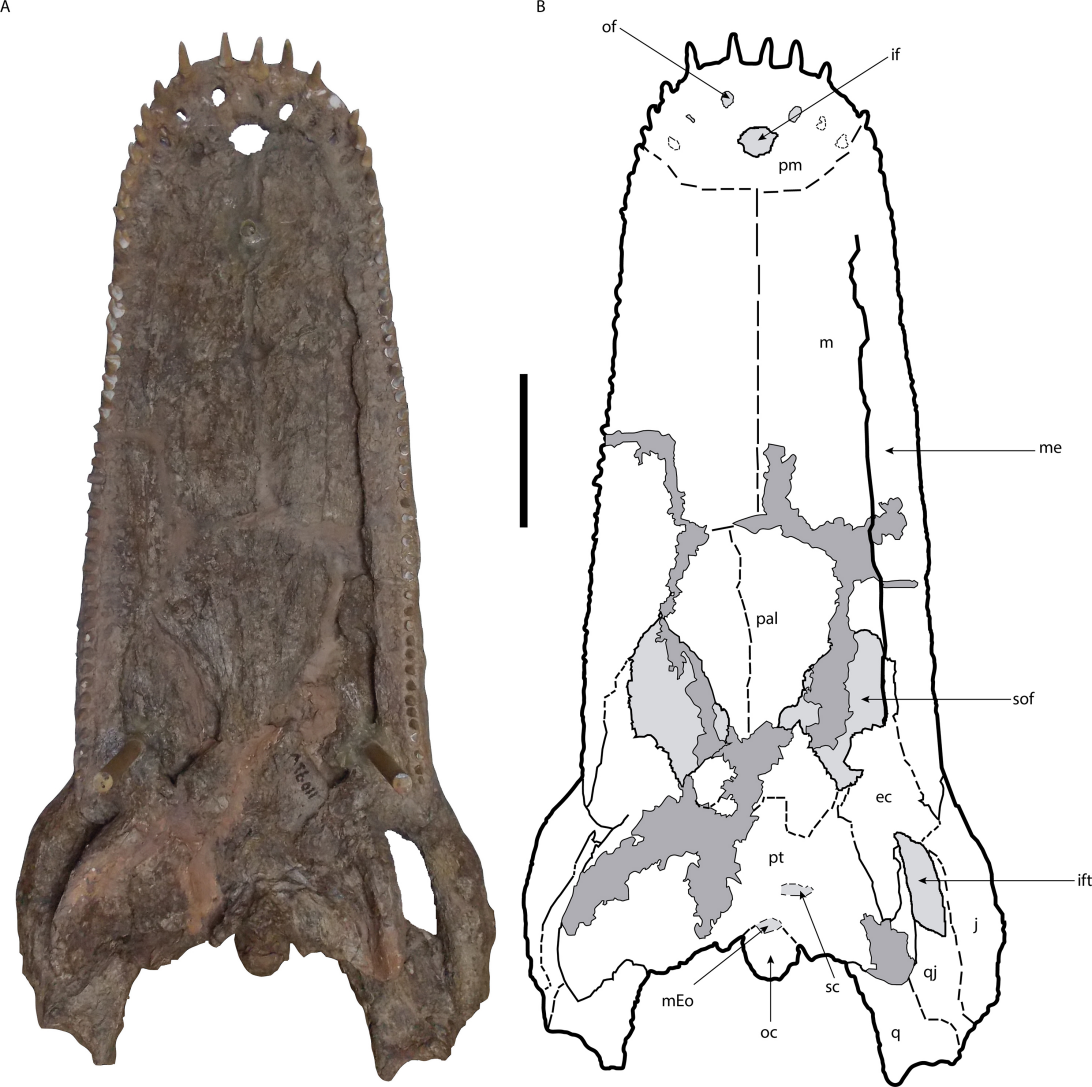
Skull of *Mourasuchus pattersoni* sp. nov. (MCNC-PAL-110-72V, holotype) in ventral view (A) with schematic drawing (B). In dark grey, the areas covered by plaster. Abbreviations: ec, ectopterygoid; if, incisive foramen; ift, infratemporal fenestra; j, jugal; m, maxilla; mEo, medial Eustachian opening; oc, occipital condyle; of, occlusal fossa; p, palatines; pm, premaxilla; pt, pterygoid; q, quadrate; qj, quadratojugal; sc, secondary choana; sof, suborbital fenestra. Scale bar equals 20 cm.

**Figure 4:**
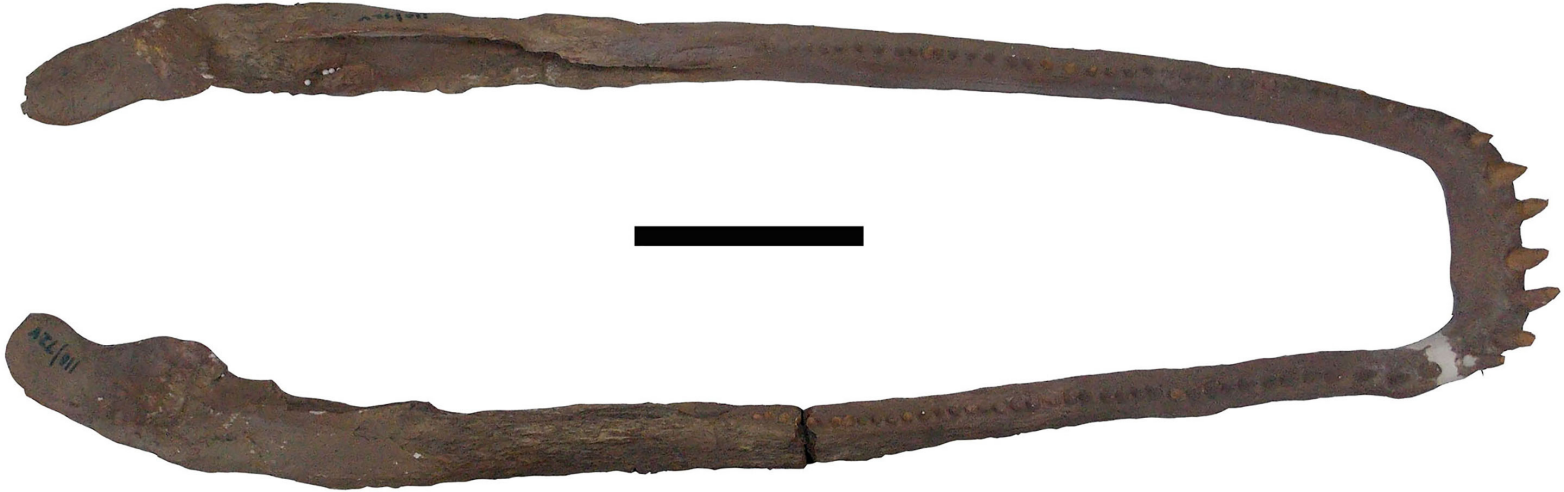
Mandibles of *Mourasuchus pattersoni* sp. nov. (MCNC-PAL-110-72V, holotype) in dorsal view. Scale bar equals 20 cm.

The restored skull and mandibles (Figs. 2–4) were returned to the Museo de Ciéncias Naturales de Caracas (MCNC) in 2002, without ever receiving a morphological description. The postcranial material described by Langston (2008), however, never underwent restoration and was not returned to Venezuela.

Prior to Langston (2008), other authors had mentioned the existence of this specimen without describing it. Medina (1976) mentioned the occurrence of *Mourasuchus amazonensis* in the Urumaco Formation. Although the author does not specify the specimen referred to, it is probable that the reference is to MCNC-PAL-110-72V, since the remains of the crocodilian described in the paper (*Melanosuchus fisheri* Medina, 1976) were collected in the same field trip as MCNC-PAL-110-72V. Additionally, Aguilera (2004) also mentioned the existence of this specimen while apparently assigning it to *Mourasuchus arendsi*, as the author then recognized this species to be the only one to occur in the Urumaco Formation. Scheyer & Delfino (2016) also tentatively suggested the assignment of the specimen to *M. arendsi*. Despite these previous taxonomic assignments, the detailed morphological study of the skull and mandibles of MCNC-PAL-110-72V performed in this paper considers this specimen a new species of *Mourasuchus* from the Urumaco Formation: *M. pattersoni* sp. nov.

MCNC-PAL-110-72V was compared especially to the other species of *Mourasuchus* through personal observations and the published literature, including the descriptions of *M. amazonensis* (Price, 1964), *M. atopus* (Langston, 1965), *M. arendsi* (Bocquentin-Villanueva, 1984) and to the specimens traditionally referred to *M. nativus* (Gasparini, 1985; Cidade et al., 2013). Among the last ones, *M. pattersoni* sp. nov. was compared especially with UFAC-1424, a partial skull associated with a fragmentary right mandibular ramus (see Bocquentin & Souza-Filho, 1990 and Bona, Degrange & Fernández, 2013, Fig. 2C) from the late Miocene Solimões Formation of Brazil that may be more closely related to *M. amazonensis* for sharing with this species the same morphology of the jugals. This taxonomic perspective of UFAC-1424 is distinct from those of the holotype and other described specimens of *M. nativus*, which consist solely of isolated skull tables (see Gasparini, 1985, Cidade et al., 2013 and Scheyer et al., 2013) and can be assigned to *M. arendsi* following Scheyer & Delfino (2016). Further studies are required to settle the taxonomic assignment of UFAC-1424; in this paper, this specimen is considered as a potential distinct form of *Mourasuchus* and was used for morphological comparisons and included in the phylogenetic analysis.

The phylogenetic analysis of this paper was performed using the software “Tree analysis using New Technology”—TNT, version 1.1 (Goloboff, Farris & Nixon, 2008), which is made available freely on the internet by the Willi Hennig Society. The analysis was made with 10,000 replications, a random seed value of “0” and 20 cladograms saved per replication. The branch swapping algorithm ‘tree-bisection-reconnection’ was selected. The morphological data matrix was scored in the software Mesquite, version 2.75 (Maddison & Maddison, 2011).

The phylogenetic analysis was performed with a modified version of the matrix of Brochu (2011), including 89 Operational Taxonomic Units (OTUs) with 88 Eusuchian taxa in the ingroup and the non-Eusuchian crocodyliform *Bernissartia fagesii* Dollo, 1883 as the outgroup. Of the taxa in the ingroup, 74 were already included in the matrix of Brochu (2011) and 13 were included from scorings available in other papers. The scoring of *Mourasuchus pattersoni* sp. nov. was made by the authors and the postcranial information was scored using Langston (2008) (see S.2. in Supplemental Information 1 for details). *Bernissartia fagesii* was also included in Brochu (2011). The analysis of this study included 187 morphological characters, with 175 from Brochu (2011), four from Salas-Gismondi et al. (2015), three from Barrios (2011), two from Bona, Riff & Gasparini (2013), one from Aguilera, Riff & Bocquentin-Villanueva (2006), one from Pinheiro et al. (2012) and one new character. Additionally, five characters were rephrased (see Supplemental Information 1 for details). The character scorings of the taxa included from previous publications had many scorings changed in the present paper based on personal observations of the specimens and on the descriptions available in the literature (see S.1. in Supplemental Information 1 for details).

The images of this contribution were modified with the use of the software Adobe Illustrator CC 2014 and Adobe Photoshop CC 2014. The photographs of the fossil were altered for the inclusion of schematic drawings and/or arrows to point to relevant structures, along with the inclusion of the structures’ abbreviations.

The electronic version of this article in Portable Document Format (PDF) will represent a published work according to the International Commission on Zoological Nomenclature (ICZN), and hence the new names contained in the electronic version are effectively published under that code from the electronic edition alone. This published paper and the nomenclatural acts it contains have been registered in ZooBank, the online registration system for the ICZN. The ZooBank LSIDs (Life Science Identifiers) can be resolved and the associated information viewed through any standard web browser by appending the LSID to the prefix http://zoobank.org/. The LSID for this publication is: urn:lsid:zoobank.org:pub:D2F1F0A2-5E2B-4111-A6E4-AC78480CF3B2. The online version of this work is archived and available from the following digital repositories: PeerJ, PubMed Central and Controlled Lots Of Copies Keeps Stuff Safe (CLOCKSS).

### Geological setting

The Urumaco Formation, an approximately 2,000 m thick lithostratigraphic unit, shows a diverse lithology made of sandstone, claystone, siltstone and limestone (Linares, 2004). It has been traditionally divided into Lower, Middle and Upper Members (Linares, 2004). The Urumaco Formation was deposited during a time that extends from the latest part of the middle Miocene to the whole late Miocene (Linares, 2004). The dominant paleoenvironment during the sedimentation of the Urumaco Formation is still unclear. According to Díaz de Gamero & Linares (1989) and Hambalek et al. (1994), the sedimentation of the Urumaco Formation occurs in a complex of marginal and near coastal environments, while Quiroz & Jaramillo (2010) suggest that the formation probably was deposited in a prograding strandplain-deltaic complex. In any case, the rich vertebrate assemblage (including fresh-water and marine vertebrates) described by several authors (e.g., Sánchez-Villagra et al., 2010; Rincón et al., 2015) documented the highly variable environments along the sedimentary sequence.

The holotype of *Mourasuchus pattersoni* sp. nov. is considered to have been collected in the Upper Member of the Urumaco Formation (see Langston, 2008), which is comprised of lutites and sandstones without calcareous elements. Its sediments are interpreted as deposited in fluvial channels and flood basins, with occasional marine influence (Linares, 2004). The fossiliferous assemblages of the Upper Member are considered to be late Miocene (Linares, 2004).

## Results

### Systematic paleontology

**Table:** 

CROCODYLIA Gmelin, 1789, *sensu* Benton & Clark, 1988
ALLIGATOROIDEA Gray, 1844 (*sensu* Norell, Clark & Hutchison, 1994)
CAIMANINAE Brochu, 2003 (Following Norell, 1988)
*Mourasuchus* Price, 1964

Type species: *Mourasuchus amazonensis* Price, 1964.

Type locality: Outcrop on the left bank of the Juruá River in the Marechal Thaumaturgo Municipality, state of Acre (see Price, 1964).

Type horizon: Solimões Formation, late Miocene, Brazil.

Included species: *M. amazonensis* Price, 1964; *M. atopus* (Langston, 1965); *M. arendsi* Bocquentin-Villanueva, 1984; *M. pattersoni* sp. nov.

Emended diagnosis: *Mourasuchus* is diagnosed by the following characteristics: dentary symphysis very short, extending only to the level of the first alveolous; orbits smaller than infratemporal fenestrae; prefrontal and frontal thickened, forming a marked knob at the anteromedial margin of the orbits; dentary linear between fourth and tenth alveoli; posterior teeth and alveoli of maxilla and/or dentary laterally compressed; nasals excluded, at least externally, from the naris, with premaxillae and nasals still in contact; dorsal premaxillary processes long, extending beyond the level of the third maxillary alveolus; frontoparietal suture linear between supratemporal fenestrae; an extremely wide, compressed and long rostrum related with a very small skull table; lateral border of rostrum without festooning; prefrontals contacting at the midline, so that nasals do not contact the frontal in dorsal view; slender U-shaped mandibles that curve from first to fifth alveoli and then are straight posteriorly to sixth alveolus; upper and lower tooth rows with more than 40 teeth; osteoderms with conspicuous spines on the dorsal surface. Emended from Price (1964), Langston (1965), Langston (1966), Bocquentin & Souza-Filho (1990), and Bona, Riff & Gasparini (2013).

Stratigraphic and geographic range: middle to late Miocene from Argentina, Bolivia, Colombia, Peru, Venezuela and the Brazilian Amazonia.

*Mourasuchus pattersoni* sp. nov.

Holotype: MCNC-PAL-110-72V, an almost complete skull with both mandibles and several post-cranial remains including atlas, axis, two sacral vertebrae and the first caudal vertebra along with tentatively identified seven cervical, six thoracic, three lumbar and 13 caudal vertebrae, five cervical ribs and an unknown number of sacral and caudal ribs along with fragments of thoracic ribs, both scapulae and coracoids, a right ilium and ischium and 15 osteoderms (see Langston, 2008).

Type locality: according to Langston (2008), the holotype was collected in a locality recorded as “3 1/2 km N 30°W of El Picacho, on the up side of the Chiguaje fault”, about 6.5 km N 24°E of the town Urumaco, Falcón state, Venezuela. There is some uncertainty about this, however—see Langston (2008, p. 126) for detailed information about this issue.

Type horizon: late Miocene, Upper Member of the Urumaco Formation.

Etymology: “*pattersoni*” is a reference to the eminent Anglo-American paleontologist Bryan Patterson (1909–1979), who is responsible for many contributions to the vertebrate paleontology of the Americas and conducted the field work in which the holotype of the species was collected.

Diagnosis: jugal both lateromedially and dorsoventrally expanded (autapomorphy) and a circular incisive foramen (autapomorphy); differs from *M. atopus* and *M. arendsi* in having an external naris wider than long; differs from *M. arendsi* and UFAC-1424 in having lateromedially constricted palatines.

### Morphological description and comparisons

#### General observations

Several portions of the skull are covered with plaster (Figs. 2–4). As such, none of these areas, detailed below, are included in the morphological description of *Mourasuchus pattersoni* sp. nov. In dorsal view, the covered surfaces include the whole skull table, most of both premaxillae, most of both nasals, partial portions of both maxillae, the majority of the posterior portion of the rostrum anteriorly to the skull table (including most of the prefrontal and lacrimal bones), most of quadrates and quadratojugals and parts of the jugals (Fig. 2). The entire surface of the left external auditory meatus and most of the right one are also covered by plaster. In ventral view, the covered surfaces include portions of the areas between the palatines and maxillae, part of the posterior part of the palatine, and some areas of the pterygoid and of the right ectopterygoid. Some parts of the dorsal roof of the suborbital fenestrae are also covered, as well as some areas of both maxillae medially adjacent to each tooth row (Fig. 3). In the mandibles, the covered regions include: all the surface of the left dentary and splenial; most of the anterior portions of the right dentary and splenial; part of the right coronoid; most of both surangulars; parts of both angulars and most of the surface of both articulars.

Artificial teeth made of plaster were also implanted in both maxillae (Figs. 2 and 3) and dentaries (Fig. 4) of *Mourasuchus pattersoni* sp. nov. No real teeth are left preserved in the holotype, precluding the study of any dental characters of this species. The maxillary artificial teeth count up to 43 in the right maxillae, and 45 in the left one, while the mandibular “teeth” count up to 42 in the right dentary and up to 39 in the left one. Although it is evidently not possible to confirm if these numbers correspond to the real quantity of teeth, this count corresponds to the amount of both maxillary and mandibular alveoli found in other *Mourasuchus* specimens, which exceed 40 (e.g., Price, 1964; Langston, 1965; Bocquentin & Souza-Filho, 1990; Bona, Riff & Gasparini, 2013).

#### Skull

**Figure 5:**
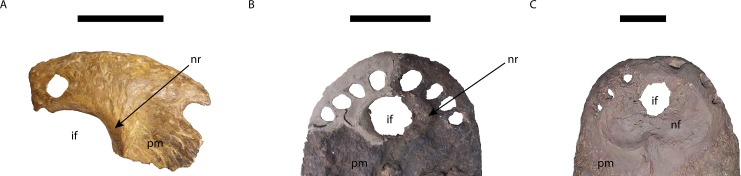
Comparison of the area of the external naris in *Mourasuchus* in dorsal view, showing the differences of the structures surrounding the incisive foramen. (A) *Mourasuchus atopus* (UCMP-38012, holotype); (B) *M. arendsi* (CIAAP-1297, holotype); (C) *M. pattersoni* sp. nov. (MCNC-PAL-110-72V, holotype). Abbreviations: if, incisive foramen; pm, premaxilla; nf, nasal fossa; nr, nasal rim. Scale bar equals 5 cm (A) and 10 cm (B and C).

**Figure 6:**
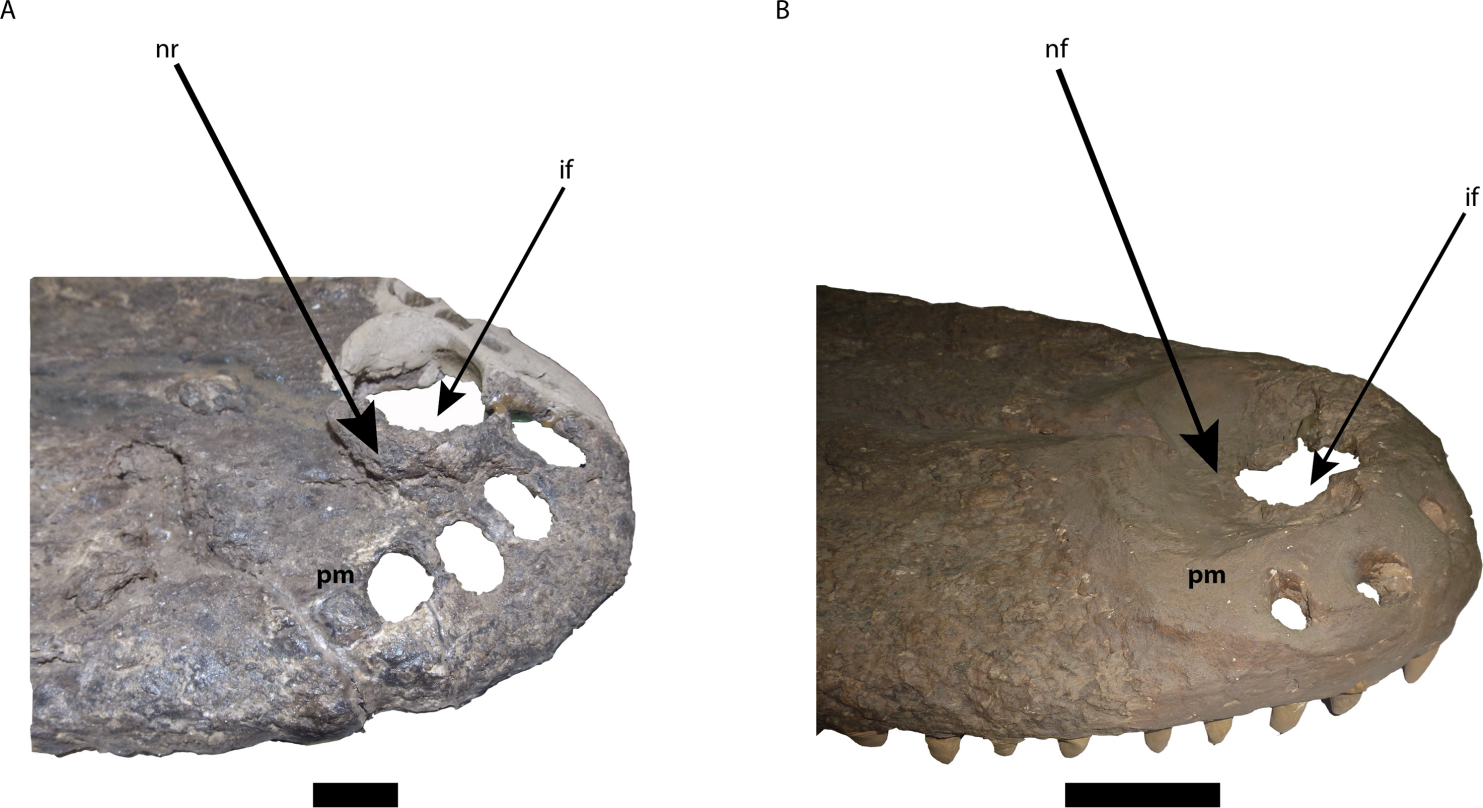
Comparison of the area of the external naris in *Mourasuchus* in right lateral view, showing the differences of the structures surrounding the incisive foramen. (A) *M. arendsi* (CIAAP-1297, holotype); (B) *M. pattersoni* sp. nov. (MCNC-PAL-110-72V, holotype). Abbreviations: if, incisive foramen; pm, premaxilla; nf, nasal fossa; nr, nasal rim. Scale bar equals 2 cm (A) and 10 cm (B).

**Figure 7:**
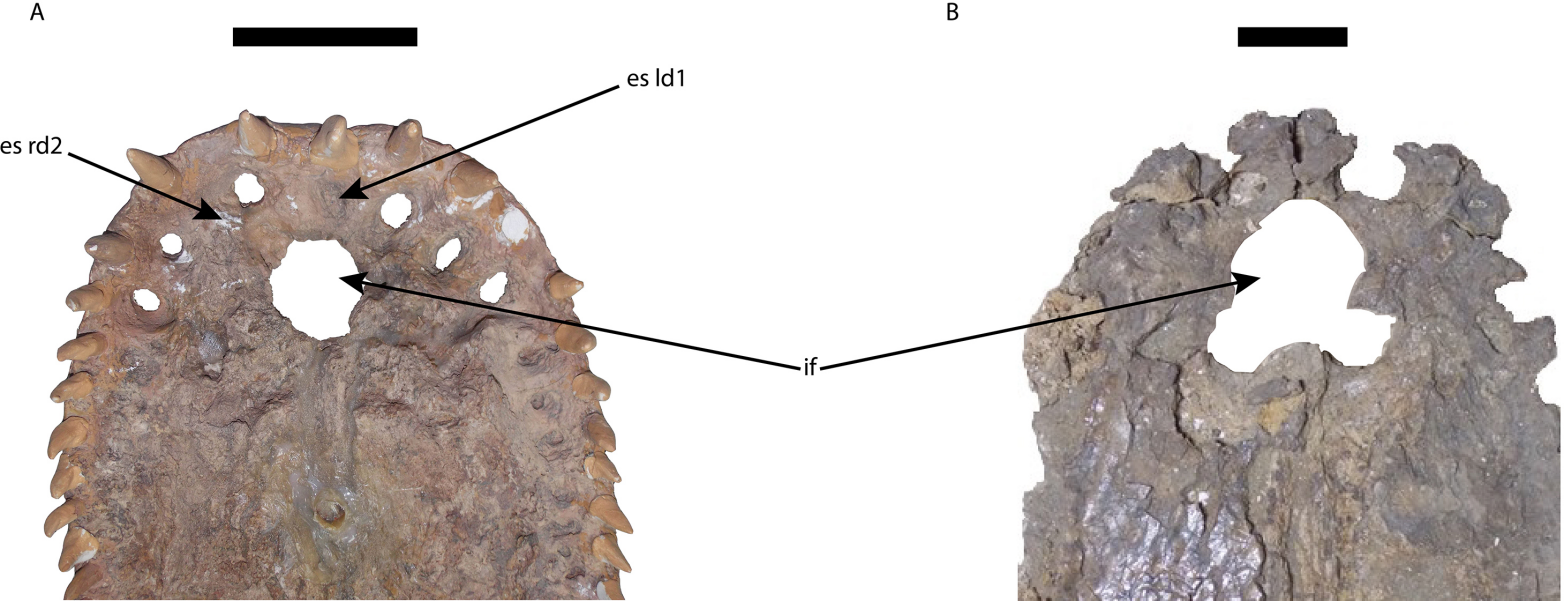
Premaxillae of *Mourasuchus* in ventral view, showing the differences in the shape of the margins of the incisive foramen. (A) *M. pattersoni* sp. nov. (MCNC-PAL-110-72V, holotype); *M. amazonensis* (DGM 526-R, holotype). Abbreviations: es ld1, erosion surface of the first tooth of the left dentary; es rd2, erosion surface of the second tooth of the right dentary; if, incisive foramen. Scale bars equal 10 cm.

The premaxillae of the holotype (MCNC-PAL-110-72V) have three perforations derived from the occlusion of mandibular teeth (Figs. 5C, 6B and 7A). This number differs from those observed in *Mourasuchus atopus* (two; see Langston, 1965), *M. arendsi* (four; see Bocquentin-Villanueva, 1984) and *M. amazonensis* (one; G Cidade, pers. obs., 2016—albeit the premaxillae of this species are very fragmented for this to be affirmed with certainity). Occlusal pits forming perforations are exhibited in several fossil and extant species of crocodylians, and are variable even within a species (e.g., *Caiman crocodilus*, Kälin, 1933; Langston, 2008). As a result, these features will not be considered as diagnostic among the species of *Mourasuchus*. Aside from these perforations, there is an eroded occlusal surface in the most medial portion of the left premaxilla, probably derived from the first left mandibular teeth (Fig. 7B). In the right premaxilla, there is also an area between the first and second visible perforations that may correspond to where the second right mandibular tooth occluded (Fig. 7B).

The external naris is large, lateromedially wider than anteroposteriorly long, occupying most of the dorsal surface of the premaxillae and forming a dorsoventrally deep fossa around the incisive foramen (Figs. 5C and 6B). This morphology is similar to the one illustrated for *Mourasuchus amazonensis* (Price, 1964), but is distinct from the small external naris seen in *M. arendsi* (Bocquentin-Villanueva, 1984; Figs. 5B and 6A). The latter taxon does not have a deep fossa, showing rather an elevated bony rim in its surface laterally to the dorsal exposure of the incisive foramen (Figs. 5B and 6A). The external naris of *M. atopus* cannot be fully observed, as this species has only part of its right premaxilla preserved (Fig. 5A). However, the elevated rim observed in *M. arendsi* may be present in *M. atopus* as well, while the presence of the deep fossa in the premaxilla of *M. atopus* cannot be confirmed (Fig. 5A). As such, while *M. arendsi* had a small, circular external naris (Character 83-0), *M. pattersoni* sp. nov. shares with *M. amazonensis* a wider than long external naris (Character 83-1). The bony rim of *M. atopus* and *M. arendsi* are treated in this study as a new state to Character 86—which is based on Character 86 of Brochu (2011)—and is absent in *M. pattersoni* sp. nov. and apparently in *M. amazonensis*, judging from the illustration of Price (1964, Fig. 1).

The incisive foramen of the holotype of *Mourasuchus pattersoni* sp. nov. can be totally observed in dorsal view, due to the large size of the external naris (Fig. 5C), similar to what is illustrated for *M. amazonensis* (Price, 1964) but different from that illustrated for *M. arendsi* (Bocquentin-Villanueva, 1984). The incisive foramen in *M. pattersoni* sp. nov. is small and circular. This condition differs from the large, tri-lobed foramen of *M. amazonensis* (Price, 1964; Fig. 7) and from the large foramen of *M. arendsi*, which is illustrated by Bocquentin-Villanueva (1984) as being lateromedially enlarged anteriorly and narrow posteriorly, resembling a “reversed-teardrop” shape. As *M. atopus* does not have its incisive foramen entirely preserved (Langston, 1965), the circular foramen of *M. pattersoni* sp. nov. may be considered as an autapomorphy of this species.

The maxillae display the same shape and size proportions as in other *Mourasuchus* species that preserve these bones (e.g., Price, 1964; Langston, 1965; Bocquentin-Villanueva, 1984; Figs. 2 and 3). Only few contacts with other bones are visible: the most posterior portion of each maxilla-nasal suture, in dorsal view; the suture with the right ectopterygoid and part of the suture with the left ectopterygoid, in ventral view (Figs. 2 and 3). Also in ventral view, both maxillae show, medially to the tooth row, an elevation that, in the left maxilla, is very prominent and platform-like. It starts arising at the level between the 10th and the 11th implanted “teeth” and keeps elevating continuously in an anteroposterior direction until the medial suture of the maxilla with the left ectopterygoid. This increased elevation present in the left maxilla may be a taphonomical issue, which may be the result of a lateromedial displacement during its post-mortem diagenesis that may have caused other deformations in the cranium, seen in ventral view. An slight elevation, more similar to the one present in the right maxilla of *M. pattersoni* sp. nov. is also seen in the left maxilla of the holotype of *M. amazonensis* (DGM 526-R).

The nasals show the same anteroposteriorly long morphology seen in other *Mourasuchus* (e.g., Price, 1964; Bocquentin-Villanueva, 1984; Fig. 2). As seen for the maxillae, most of the contacts of the nasals with other bones are not visible. It is not possible to confirm if the nasals contact the external naris externally in *M. pattersoni* sp. nov. In most caimanines, the nasals reach the external naris but do not bisect it (Brochu, 1997). In *Mourasuchus* specimens with this feature preserved, the nasals contact the premaxillae but not the external naris, as in *M. amazonensis* (Price, 1964) and *M. arendsi* (Bocquentin-Villanueva, 1984). This feature corresponds to State 2 of Character 82 of Brochu (2011). The suture between the nasals is only visible in part of its posteriormost portion (Fig. 2), practically at the same level where the suture of each nasal with its respective maxilla is evident. The suture of the right nasal with the right prefrontal is visible only at the anteriormost portion.

All other contacts of both prefrontals, besides the one with the right nasal, are not visible in *Mourasuchus pattersoni* sp. nov. (Fig. 2). A similar situation is seen for the lacrimals and jugals in dorsal view, for which there are only weakly marked lines in the fossil or the contacts are simply covered by plaster (Fig. 2). The inferred limits of these bones indicate that the lacrimals of the holotype of *M. pattersoni* sp. nov. do not contact the nasals, although this cannot be confirmed, which would be similar to what is figured for *M. amazonensis* (Price, 1964) and *M. arendsi* (Bocquentin-Villanueva, 1984). This morphology is also seen in *Purussaurus*—such as *P. neivensis* (Mook, 1941) and *P. mirandai* Aguilera, Riff & Bocquentin-Villanueva, 2006 (Langston, 1965; Aguilera, Riff & Bocquentin-Villanueva, 2006). A new state to Character 128 of Brochu (2011) was proposed by Aguilera, Riff & Bocquentin-Villanueva based on this morphology (see ‘Phylogenetic analysis’ and Supplemental Information 1 for details).

**Figure 8:**
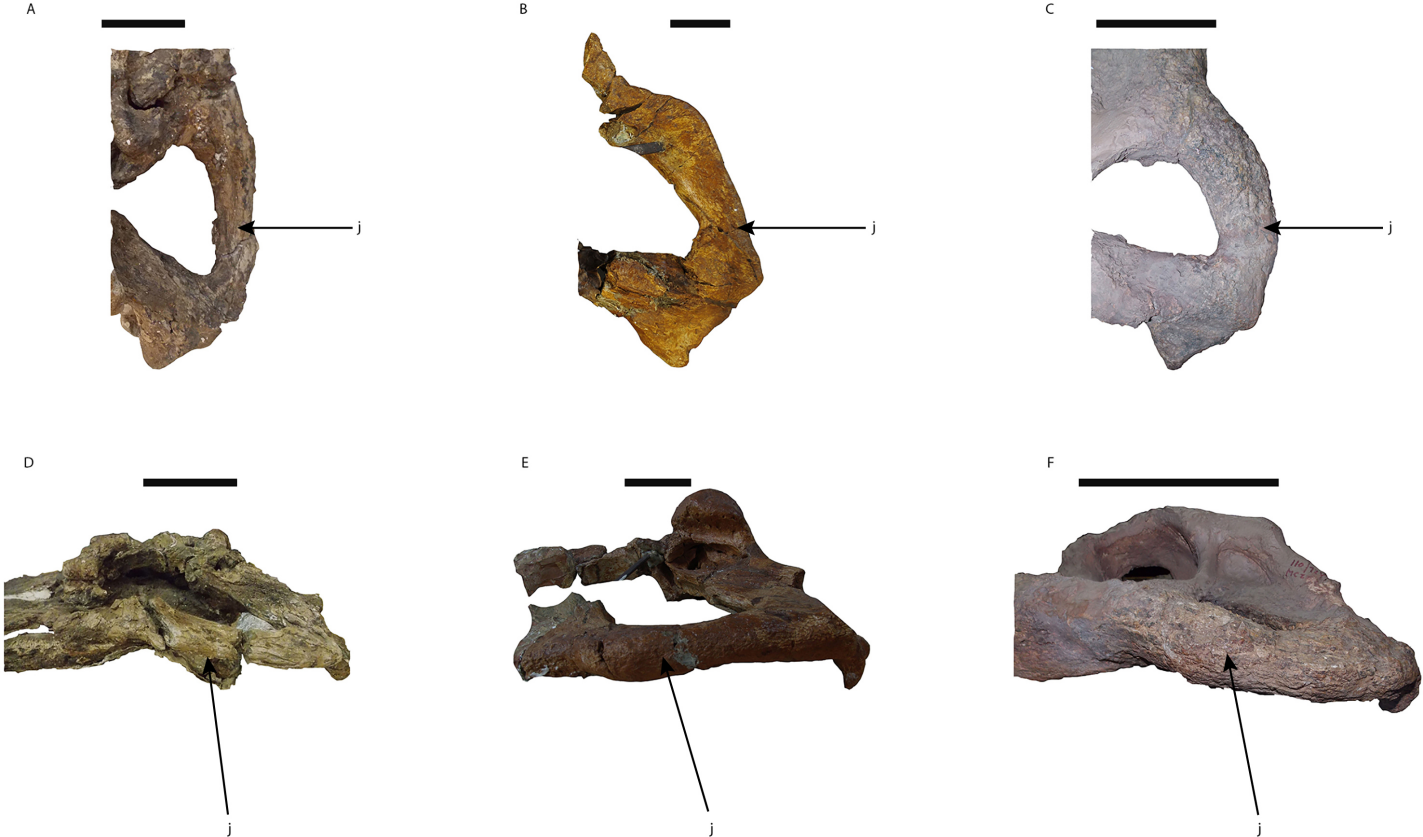
Morphological disparity of the jugals in *Mourasuchus*. *M. arendsi* (CIAAP-1297, holotype) in dorsal (A) and lateral (D) views; UFAC-1424 in dorsal (B) and lateral (E) views; *M. pattersoni* sp. nov. (MCNC-PAL-110-72V, holotype) in dorsal (C) and lateral (F) views. Abbreviations: j, jugal. Scale bar equals 10 cm.

Lateral to the skull table on both sides, the quadrate, quadratojugal and jugal bones are present. In this region, only the suture between the left quadratojugal and jugal, in dorsal view, and part of that between the right quadratojugal and the jugal, in ventral view, are evident. The jugals of *Mourasuchus pattersoni* sp. nov. are completely preserved. These bones are robust, showing a cylindrical, lateromedially wide and dorsoventrally high shape (Figs. 8C and 8F). This morphology differs from those of *M. atopus* and *M. arendsi*, which possess lateromedially slender, dorsoventrally low jugal bones (Figs. 8A and 8D), and from *M. amazonensis* and UFAC-1424, which have lateromedially wide, but dorsoventrally flattened jugals (Figs. 8B and 8E). In this way, the jugal morphology seen in *M. pattersoni* sp. nov. is unique to this taxon and therefore may be considered as an autapomorphy.

**Figure 9:**
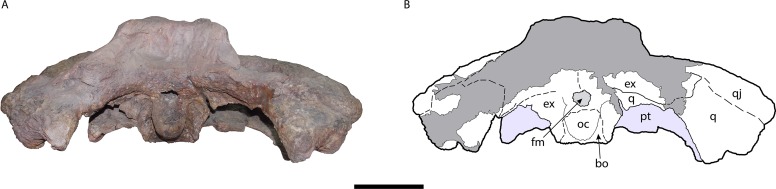
Occipital area of the skull of *Mourasuchus pattersoni* sp. nov. (MCNC-PAL-110-72V, holotype; (A) with schematic drawing (B). In dark grey, the areas covered by plaster. Abbreviations: bo, basioccipital; ex, exoccipital; fm, foramen magnum; oc, occipital condyle; pt, pterygoid; q, quadrate; qj, quadratojugal. Scale bar equals 10 cm.

On both sides, the quadratojugal has an anterior process medial to the jugal—the process “along the lower temporal bar” of Brochu (2011, Character 144-0; Character 143-0 in the present paper), a common morphology in Alligatoroidea (Brochu, 1997; Brochu, 2011). This feature can also be seen in ventral view, on both sides. On the right side of the skull, however, the ventral exposure of the anterior process of the quadratojugal is very extensive, comprising almost the whole of the ventrolateral margin of the infratemporal fenestra. This extension is anteroposteriorly longer than those observed in some caimanines (e.g., Carvalho, 1951; Medem, 1981; Medem, 1983; Brochu, 1999) including *M. arendsi* (Bocquentin-Villanueva, 1984), but is similar to the holotype of *M. amazonensis* (DGM 526-R). The presence of this morphology in both *M. pattersoni* sp. nov. (Figs. 2 and 3) and *M. amazonensis* is here interpreted either as being a natural feature of these specimens that can be seen as a synapomorphy of *M. pattersoni* and *M. amazonensis* or as a character correlated to the lateromedial expansion of the jugal present in both species. There is also the possibility, however, that this morphology may have been enhanced or entirely caused by taphonomic distortion: a ventral displacement of the anterior process of the quadratojugal that made it more broadly exposed on the ventral side of the skull. Future studies on this character in *Mourasuchus* may settle these issues. The quadrates are almost fully preserved, with only slight damage in the medial hemicondyle of the left quadrate and in the lateral hemicondyle of the right quadrate. Such damages do not allow to determine which of the hemicondyles is the largest (Character 180). Only the suture of the right quadrate with the right exoccipital is evident (Fig. 9) and the anterolateral limits of both quadrates are covered by plaster (Fig. 2). The presence of the foramen aereum is not evident in either of the quadrates. The dorsal surface of the quadrates lacks a proeminent, mediolaterally thin crest (Character 178-0). The ventral surface bears crests, and not a prominent knob, for attachment of the posterior mandibular adductor muscle (Character 179-0). These last two characters are shared with most Eusuchia (Brochu, 2011).

The supraoccipital and squamosal surfaces that are exposed in posterior (occipital) view are entirely covered with plaster. Most of the surface of both exoccipitals are in the same situation, except for the ventralmost portions and the paroccipital processes (Fig. 9). The ventral sutures of both exoccipitals with the basioccipital are not evident. The basioccipital is fully preserved, including the occipital condyle (Fig. 9). The medial crest of the basioccipital is preserved; this structure serves as the attachment point for the muscles *M. basioccipitovertebralis* and *M. occipitotransversalis profundus* in Crocodylia (Iordansky, 1973). In ventral view, between the basioccipital and the pterygoid, the opening of the medial Eustachian tube is discernible (Fig. 3), while the openings of the lateral Eustachian tubes are not.

The palatines of *Mourasuchus pattersoni* sp. nov. are almost fully preserved (Fig. 3). The right suborbital fenestra has all of its margins preserved, while the left fenestra has the anterior end eroded. The sutures of the palatines with the maxillae are not evident. The suture between the palatines and the pterygoid are not fully evident, but could be represented as in Fig. 3. The area between the left palatine and the pterygoid is distorted. It suffered a ventromedial displacement probably due to the taphonomic effects possibly related to the medial displacement that formed the distortion present in the left maxilla. The palatines exhibit an accentuated lateromedial constriction in their anteroposteriorly most medial portions (Fig. 3). This morphology is shared with *M. atopus* (see Langston, 1965), but is different from those of *M. arendsi* (Bocquentin-Villanueva, 1984) and UFAC-1424 (Bocquentin & Souza-Filho, 1990), whose palatines are lateromedially expanded. This feature could not be observed in *M. amazonensis*, since Price (1964) does not provide any illustration of the ventral view of the skull, and this area of the holotype (DGM 526-R) is eroded (G Cidade, pers. obs., 2016).

The ectopterygoids are completely preserved, forming most of the lateral margins and the lateral portions of the posterior margins of the suborbital fenestrae (Fig. 3). The suture of the left ectopterygoid with the jugal is not evident, whilst the suture with the pterygoid is visible except for parts of its most anterior portion. This suture exhibits a flexure (Brochu, 2011, Character 126-0), a morphology present in most Caimaninae (Brochu, 2011; Salas-Gismondi et al., 2015) (Fig. 3). The suture of the left ectopterygoid with the maxilla can only be observed in its most anterior and posterior portions; in between, the suture is covered by a resin used to fix a wooden stick into the specimen during restoration. The left ectopterygoid is also slightly medially displaced, which can be another consequence of the aforementioned taphonomic medial displacement suffered by the left side of the skull. The suture of the right ectopterygoid with the jugal can be fully observed, exhibiting an oblique shape. The suture with the maxilla is only obscured on the most anterior part, while the suture with the pterygoid is covered by plaster. In both ectopterygoids, it is possible to see both the maxillary and the descendent (which articulates posterodorsally with the pterygoid) processes of the ectopterygoid. The descendent process does not extend to the posterior tip of the lateral pterygoid flange, a common morphology in Eusuchian crocodyliforms (Brochu, 2011).

The pterygoid of *Mourasuchus pattersoni* is almost completely preserved. It is not possible to observe with accuracy whether the pterygoid of the holotype reached the posterior end of the suborbital fenestra, as described for all Neosuchian crocodyliforms except *Caiman yacare* (Brochu, 1997; Barrios, 2011; Fig. 3). The surface of the pterygoid exposed in ventral view is eroded, and as a result the complete outline of the secondary choana is not discernible. Only a perforation in the posteromedial portion of the pterygoid can be seen, and may correspond to the left half of the secondary choana of the holotype of *M. pattersoni* sp. nov. (Fig. 3). This structure has a nearly rectangular-shape similar to the secondary choana of *M. atopus* (see Langston, 1965, Fig. 28). Medial to this perforation, there is a bony formation that may correspond to the choanal septum that occurs in most Alligatoridae (Brochu, 1997). The assignment of these two features to these respective structures, however, must be seen with caution until better preserved material of *M. pattersoni* is eventually described.

#### Mandibles

**Figure 10:**
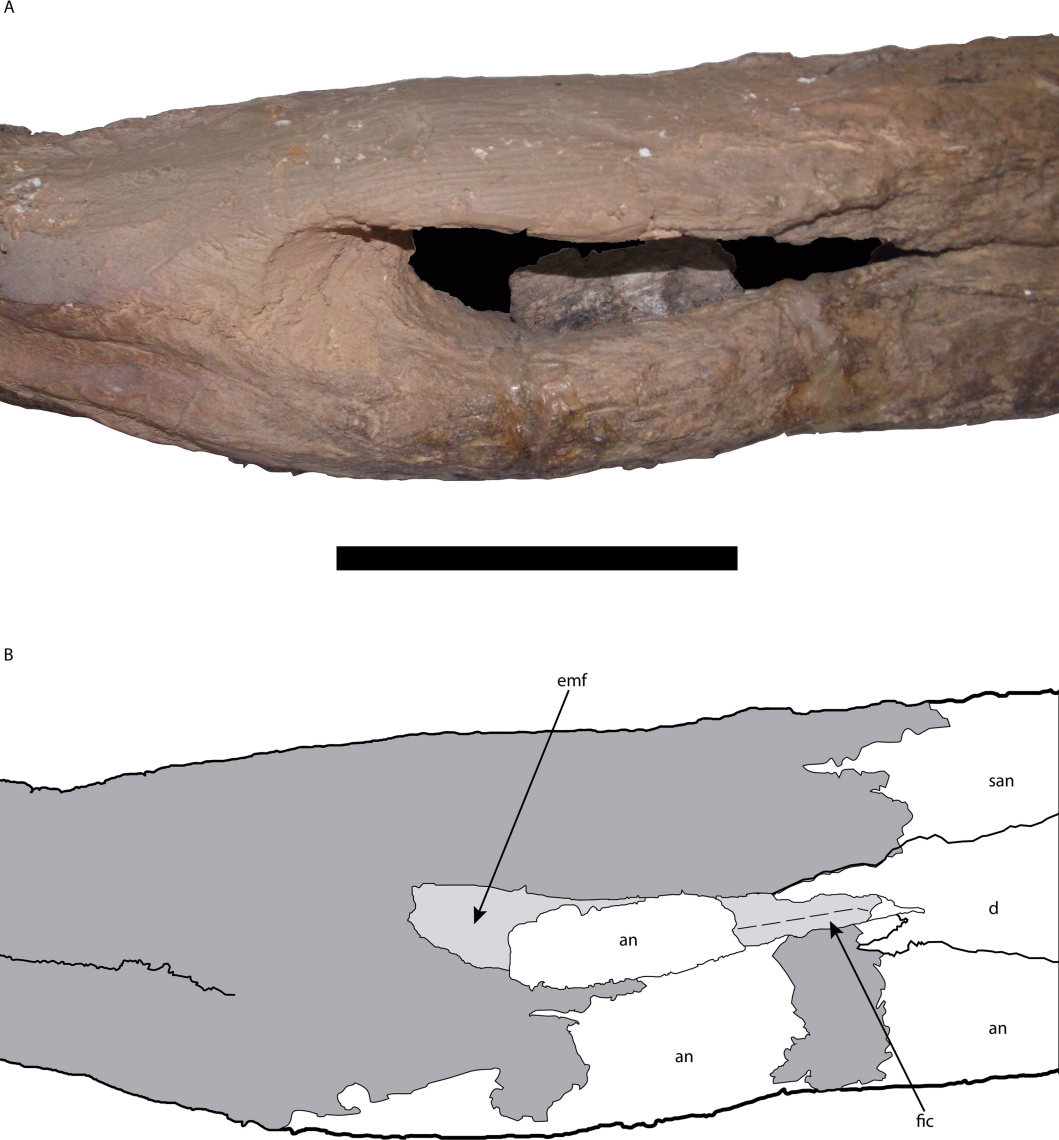
Posterior portion of the right mandible of *Mourasuchus pattersoni* sp. nov. (MCNCPAL- 110-72V, holotype) in lateral view (A) with schematic drawing (B). In dark grey, the areas covered by plaster. Abbreviations: an, angular; d, dentary; emf, external mandibular fenestra; fic, foramen intermandibularis caudalis; san, surangular. Scale bar equals 5 cm.

Both dentaries are heavily modified by the restoration (Fig. 4). Generally, the dentaries of *M. pattersoni* are similar to those of other species of *Mourasuchus*, having a “U-shaped” form that is curved from the first to the fourth alveolous and that becomes linear posterior to the fourth alveolous (Character 50-0). In lateral view, the right dentary is sutured with the surangular dorsally and with the angular ventrally, whilst comprising the anterior portions of both the dorsal and ventral margins of the external mandibular fenestra (Fig.  10). In medial view, however, the contact with the splenial is not distinguishable. In the left dentary, none of these contacts can be seen due to the plaster covering.

**Figure 11:**
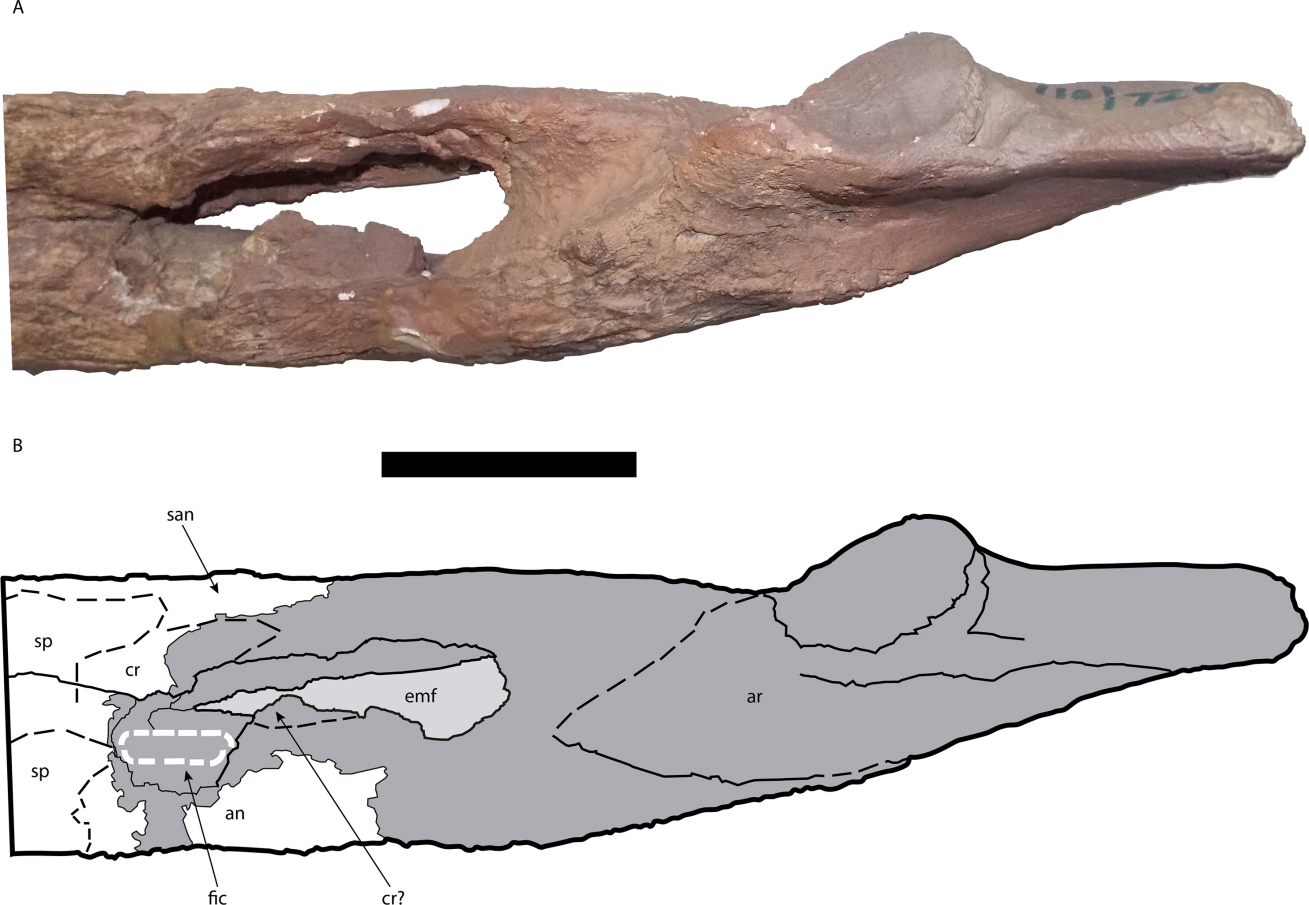
Posterior portion of the right mandible of *Mourasuchus pattersoni* sp. nov. (MCNC-PAL- 110-72V, holotype) in medial view (A) with schematic drawing (B). In dark grey, the areas covered by plaster. Abbreviations: an, angular; ar, articular; cr, coronoid; emf, external mandibular fenestra; fic, foramen intermandibularis caudalis; san, surangular; sp, splenial. Scale bar equals 10 cm.

Both splenials are only partially preserved. In the left splenial, the preserved surface is entirely covered by plaster, whilst the right one is almost completely uncovered. It forms the medial surface of the mandibular ramus as typical in Crocodylia, while being anteroposteriorly expanded in accordance with the distinct mandibular morphology of *Mourasuchus*. The foramen intermandibularis oralis (see Iordansky, 1973 and Brochu, 1997, Character 49) is not evident. As the contacts between the splenial and the dentary are not distinguishable anteriorly, it is not possible to assert where the anteriormost point of the splenial is. The suture of the right splenial with the dentary is only evident at its most posterior portion, throughout the level of the last two implanted “teeth” of the dentary (Fig. 11). The surface of the right splenial is preserved only until this level. Posteriorly, the splenial preserves only a dorsal fragment and a ventral fragment, whilst the surface of the bone that would be between them is absent. The dorsal fragment contacts the surangular dorsally, and the coronoid posteriorly. The ventral fragment contacts the angular posteriorly.

The left coronoid is not preserved. The right coronoid preserves a dorsal portion that anteriorly contacts the posterior area of the dorsal region of the splenial and also dorsally contacts the surangular (Fig. 11). Aside from this, it is possible that the part of the posteroventral portion of the coronoid is also preserved. In medial view, the ventral area of the adductor fossa has a fragment of bone positioned dorsal to the ventral margin of the angular. It is interpreted here that the coronoid is preserved at the anterior portion of the dorsal margin of this fragment, while the angular comprises the rest of the fragment (Fig. 11). This interpretation, however, is tentative. In between these two possibly preserved portions, the right coronoid is absent.

**Figure 12:**
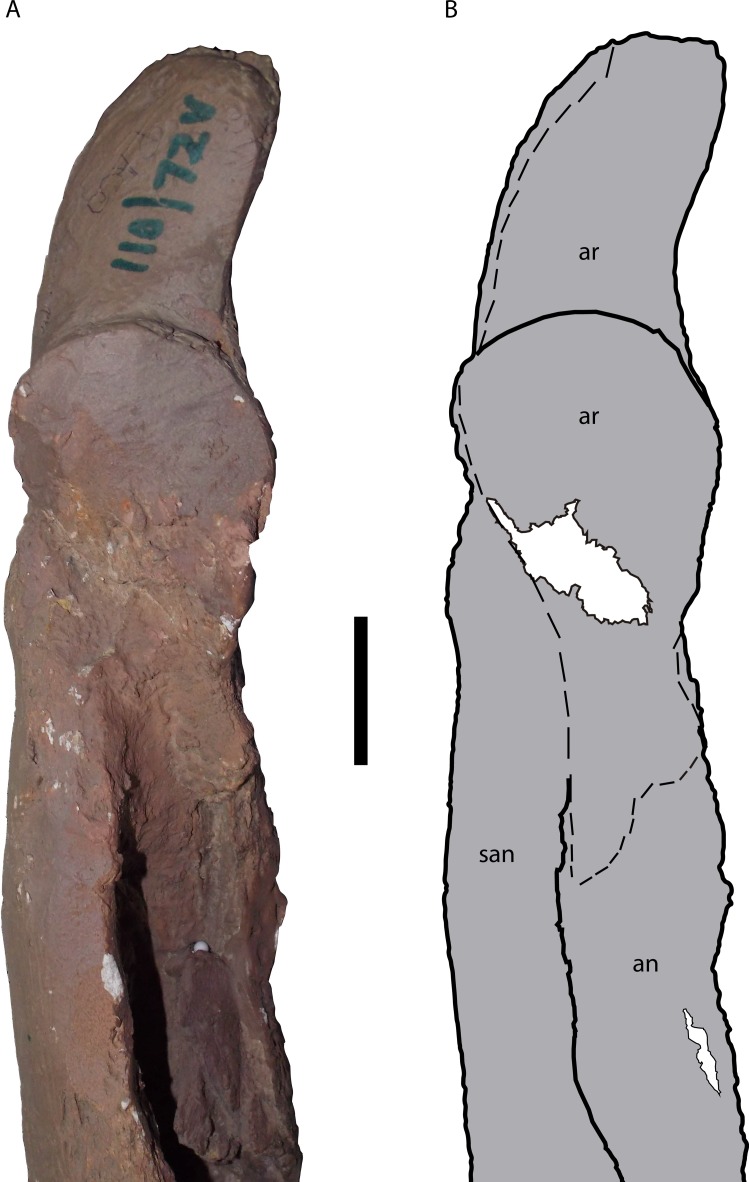
Posterior portion of the right mandible of *Mourasuchus pattersoni* sp. nov. (MCNC-PAL-110-72V, holotype) in dorsal view (A) with schematic drawing (B). In dark grey, the areas covered by plaster. Abbreviations: an, angular; ar, articular; san, surangular. Scale bar equals 5 cm.

Both surangulars are partially preserved. They form the lateral part of the most posterior portion of the mandibular rami, as it is common in Crocodylia, and comprise the dorsal margin and the dorsal portion of the posterior margin of the external mandibular fenestra (Figs. 10– 12). The left surangular is almost completely covered by plaster, while a portion of its medial surface, in the dorsal margin of the adductor fossa, is absent. Only three parts of the left surangular are not covered by plaster. One is the anteriormost portion of the surangular visible in both medial and dorsal views, which contacts the dentary anteriorly. The second is a portion that comprises from the posterior margin of the external mandibular fenestra, in medial view, up to the posterior contacts of the surangular with the articular (dorsally) and the angular (ventrally). The last is a portion that, in lateral view, comprises a surface from the posterodorsal end of the external mandibular fenestra up to the most anterior region of the contact of the surangular with the retroarticular process of the articular. In lateral view, the surangular-angular suture contacts the external mandibular fenestra at its posterior angle (Character 60-0), a morphology also seen in *M. atopus* and UFAC-1424. This character could not be observed in *M. arendsi* and is not preserved in *M. amazonensis*. The right surangular is also almost completely covered by plaster while some portions are absent, especially in the region located anteriorly to the dorsal margin of the adductor fossa, in medial view (Fig. 11). The parts of the right surangular not covered by plaster include its most anterior portion, where the surangular contacts the splenial and the coronoid medially, and the dentary laterally and dorsally. The only evident sutures of the right surangular are laterally with the dentary, but only in its most posterior position, and a fraction of the ventral suture with the angular, in lateral view located slightly posteriorly to the external mandibular fenestra (Fig. 10). The angular-surangular suture anterior to this point is covered by plaster, where it would contact the fenestra (Fig. 10). It is not possible to observe whether the surangular extended to the posterior end of retroarticular process (Character 72) as most posterior portions of both surangulars, as well as both retroarticular processes of the articulars, are covered by plaster (right surangular and right articular are shown in Fig. 12).

In the right mandible, it is possible to observe that the external mandibular fenestra is large to the point of allowing the foramen intermandibularis caudalis to be visible in lateral view (Fig. 10). Additionally, the surangular-dentary suture intersects the external mandibular fenestra anteriorly to the posterodorsal corner of the latter (Character 64-0; Fig. 10).

The angulars are both partially preserved, forming the ventral, medial and lateral areas of the most posterior portion of both mandibular rami. The left angular, in medial view, preserves a fragmented portion positioned medial to the adductor fossa. Slightly posterior to this fragment, the angular is completely preserved from a point located posterior to the adductor fossa until its extension that contacts the articular dorsally, reaching up to the posterior end of the articular-surangular suture in medial view. In lateral view, the left angular is almost completely covered by plaster, except for a portion immediately posterior to the external mandibular fenestra. In this exposed surface, the dorsal suture of the angular with the surangular can be seen from the point where the suture meets the external mandibular fenestra until reaching the anteriormost portion of the retroarticular process. The right angular is almost completely preserved in medial view (Fig. 11), with some absent portions at its dorsal margin, along the ventral margin of the adductor fossa. In medial view, it contacts the splenial anteriorly, possibly the coronoid dorsally, the articular posterodorsally and the surangular laterodorsally (Fig. 11). The anterior margin of the angular meets the anterior end of the ventral margin of the foramen intermandibularis caudalis (Fig. 11). In dorsal view, the right angular is also preserved except for a small portion that would be situated anterior to where the descending process of the articular bone would be, as most of the anterior portion of this structure is also not preserved (Fig. 12). In lateral view, the bone is completely preserved, contacting the surangular posterodorsally and the dentary anterodorsally (Fig. 10).

Both articular bones are present, forming most of the posterior portion of both mandibular rami. Despite extensive plaster coverings in both bones, it is possible to observe the presence of the descending process, the glenoid fossa and the retroarticular process in the two bones. Finer details about this structures, however, cannot be assessed. Also in both articulars, the most anteroventral portion of the descending process is absent, of which that of the right articular is slightly more eroded than that of the left. The left articular is almost completely covered by plaster, except for a small area ventral to the retroarticular process (which contacts the angular ventrally, in medial view) and the dorsal surface of its descending process in dorsal view. The right articular is in a similar situation, with the only uncovered part of its surface being part of the most anterior portion of its glenoid fossa (Fig. 12). The articular foramen aereum is not evident in either of the articulars. The restored retroarticular processes of *M. pattersoni* sp. nov. exhibit larger anteroposterior length and lateromedial width relative to the glenoid fossa than those of the other *Mourasuchus* species that preserve this feature: *M. atopus* (Langston, 1965, Figs. 21–23) and *M. arendsi* (CIAAP-1297, G Cidade, pers. obs., 2014). However, whether the morphology of *M. pattersoni* sp. nov. (Fig. 12) is an artificial feature caused by the restoration or a real natural characteristic cannot be affirmed until the real osteological morphology of this process is assessed.

### Phylogenetic analysis

**Figure 13:**
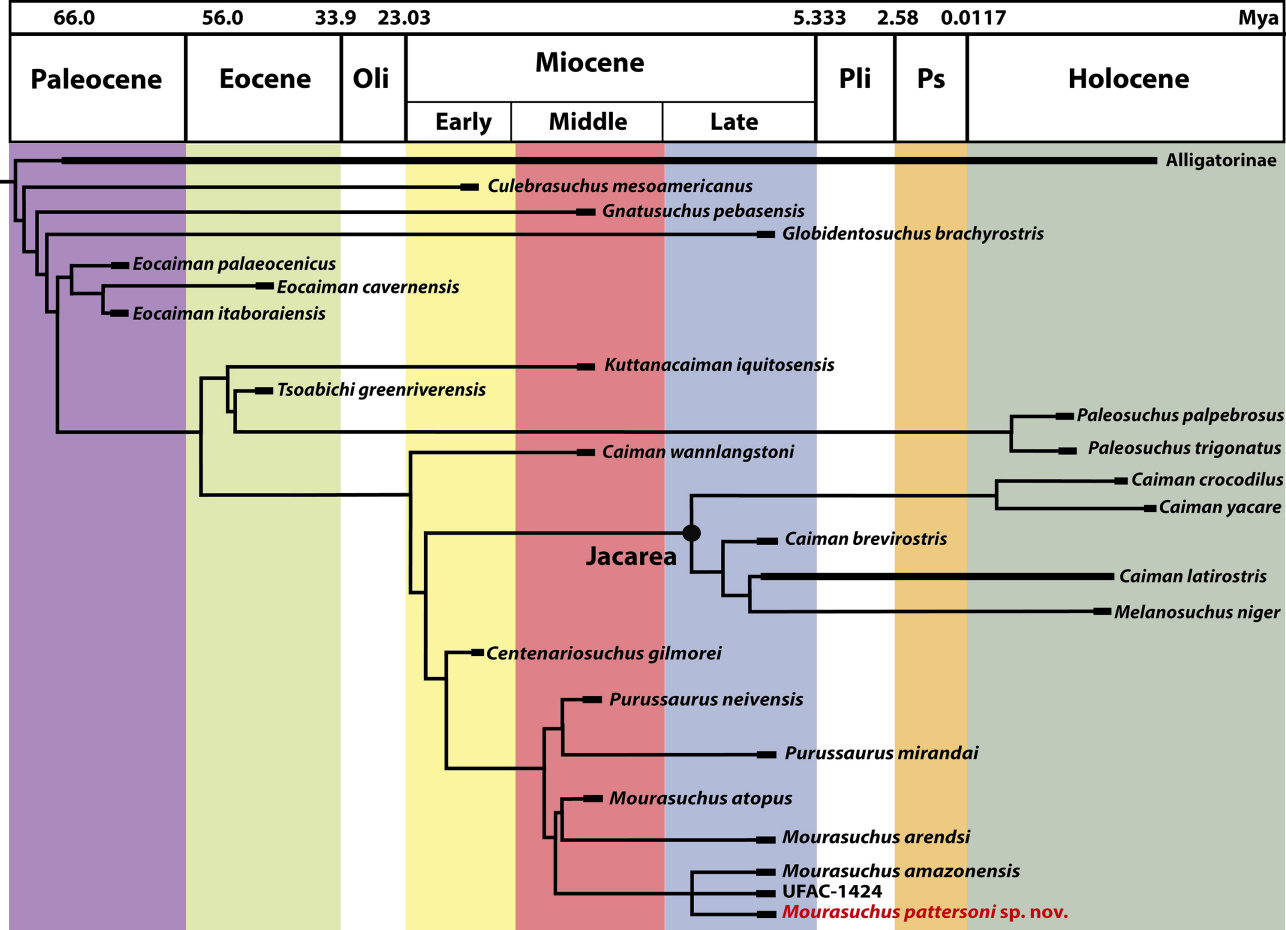
The strict consensus of 120 equally most parsimonious cladograms (629 steps; ensemble CI = 0.391; ensemble RI = 0.810) of the phylogenetic analysis performed in this paper. Only the clade Alligatoridae is shown (for a complete cladogram, see Fig. S1) exhibiting the detailed topology of Caimaninae with *Mourasuchus pattersoni* sp. nov. highlighted in red. Abbreviations: Mya, millions of years; Oli, Oligocene; Pli, Pliocene; Ps, Pleistocene.

The analysis obtained a strict consensus of 120 most parsimonious cladograms (Fig. 13 and Fig. S1) with 629 steps, an ensemble consistency index of 0.391 and an ensemble retention index of 0.810.

*Culebrasuchus*, *Gnatusuchus* and *Globidentosuchus* were recovered as successive sister-taxa to all other caimanines. All these taxa had been recovered as the basal-most caimanine in the phylogenies of their original descriptive publications—respectively Hastings et al. (2013), Salas-Gismondi et al. (2015) and Scheyer et al. (2013). The position of *Culebrasuchus* as the basal-most caimanine differs from that of Salas-Gismondi et al. (2015), which recovered this taxon as an alligatorine, within the species of *Alligator*. Also, *Culebrasuchus* and *Globidentosuchus* are not recovered as caimanines in the strict consensus of Hastings, Reisser & Scheyer (2016), in which they appear as part of a basal polytomy in the Alligatoridae clade. Synapomorphies that support the Caimaninae clade in the present analysis, with *Culebrasuchus* as the basal-most taxon, are the third maxillary alveolous as the largest one in the maxilla (Character 93-0) and supraoccipital exposure large such that the parietal is excluded from posterior edge of table (Character 159-3). In the following node, the clade that unites all Caimaninae except for *Culebrasuchus* is supported by the following synapomorphies: dermal bones of skull roof overhanging rim of supratemporal fenestra near maturity, with the fenestrae being small, with a circular or nearly circular shape (Character 151-1); medial parietal wall of supratemporal fenestrae bearing foramina (Character 153-1); exoccipitals sending slender processes ventrally to basioccipital tubera (Character 175-2); anterior extremity of the frontal short, not reaching the anterior margins of the orbits (Character 184-1). Furthermore, the node that united all Caimaninae except for *Culebrasuchus* and *Gnatusuchus* is supported by the surangular–angular suture lingually meeting the articular dorsal to the ventral tip of the articular (Character 66-1) and by the prefrontals meeting medially (Character 128-1).

More derived than these three basal taxa, *Eocaiman* appears as the sister-taxon to crown-group caimans. This clade is supported by having the splenial excluded from the mandibular symphysis, with the anterior tip of the splenial passing dorsal to the Meckelian groove (Character 54-2) as a synapomorphy. The topology among the three species of this genus is similar to that of Pinheiro et al. (2012), showing *E. palaeocenicus* Bona, 2007 as the sister-taxon of a clade formed by *E. cavernensis* Simpson, 1933 and *E. itaboraiensis* Pinheiro et al., 2012.

The crown-group caimans (see Brochu, 1999) is divided into two clades. The first one has *Kuttanacaiman* and *Tsoabichi* as successive sister-taxa to the two living species of *Paleosuchus*. *Kuttanacaiman*’s position in this clade differs significantly from Salas-Gismondi et al. (2015), which recovered this species as the sister-taxon of a two-lineage clade formed by the crown-group caimans and a clade uniting *Purussaurus* and *Mourasuchus*. This placement of *Kuttanacaiman* is supported by the following synapomorphies: lateral edges of palatines with lateral process projecting from the palatines into the suborbital fenestrae (Character 116-1) and a linear frontoparietal suture between the supratemporal fenestrae (Character 150-1). *Tsoabichi*’s position as sister-taxon to *Paleosuchus* has been recorded in other analyses (e.g., Scheyer et al., 2013; Fortier et al., 2014), and in the Adams consensus of Brochu (2010). The Palaeocene caimanine *Necrosuchus* was also recovered by Salas-Gismondi et al. (2015) as closely related to *Tsoabichi* and *Paleosuchus*, forming a polytomous clade with these two taxa. *Necrosuchus* was left out of this analysis due to its fragmentary nature and tendency to collapse the consensus. Future analyses, however, should test thoroughly whether these three taxa form a clade and which would be the most supported topology among them.

The second clade of the crown-group caimans has *Caiman wannlangstoni* Salas-Gismondi et al., 2015 as the sister-taxon of a clade with two lineages: one composed by Jacarea (*sensu* Brochu, 1999), and the other by *Centenariosuchus* as the sister-taxon of the clade containing *Purussaurus* and *Mourasuchus*. This position of *C. wannlangstoni* is supported by only one synapomorphy (the presence of the preorbital crest, Character 186-1) and differs from that recovered by Salas-Gismondi et al. (2015), in which this taxon is recovered within Jacarea. This latter clade itself is composed in this analysis by two lineages: one including *C. crocodilus* and *C. yacare* and the other having *C. brevirostris* Souza-Filho, 1987 as the sister-taxon of *C. latirostris* and *Melanosuchus niger*. The split between one clade formed by *C. crocodilus* and *C. yacare* and another formed by *C. latirostris* and *M. niger* (and other related taxa) has been recovered in many previous analyses (e.g., Brochu, 1999; Brochu, 2010; Brochu, 2011; Aguilera, Riff & Bocquentin-Villanueva, 2006; Bona, 2007; Hastings et al., 2013; Scheyer et al., 2013; Hastings, Reisser & Scheyer, 2016). *Caiman brevirostris*, on the other hand, was only included in phylogenetic analyses by Fortier et al. (2014), which recovered this taxon in a similar position to the present analysis, and by Salas-Gismondi et al. (2015), which only recovered it in a polytomy within the Jacarea clade.

The placement of *Centenariosuchus* as the sister-taxon of the clade containing *Purussaurus* and *Mourasuchus* is supported by one synapomorphy: maxilla having a linear medial margin adjacent to the suborbital fenestra (Character 111-0). This topology is different from earlier phylogenies proposed. In Hastings et al. (2013) and in Hastings, Reisser & Scheyer (2016) this taxon was recovered in the strict consensuses only as a member of a polytomy within the more derived Caimaninae, while in Salas-Gismondi et al. (2015) it was recovered as the sister-taxon of Jacarea. The sister-taxa placement of *Purussaurus* and *Mourasuchus* recovered in our analysis is similar to that recovered by Salas-Gismondi et al. (2015), which is mainly due to the exclusion of the North American Eocene form *Orthogenysuchus olseni* from both analyses. This taxon had been recovered as the sister-taxon of *Mourasuchus* in many previous analyses of Caimaninae (e.g., Brochu, 1999; Aguilera, Riff & Bocquentin-Villanueva, 2006; Bona, 2007; Hastings et al., 2013; Scheyer et al., 2013; Fortier et al., 2014), but according to Salas-Gismondi et al. (2015) ongoing preparation of the holotype (and only known specimen) of *Orthogenysuchus* revealed significant changes in the scoring of the characters for this taxon that have not been published yet. As a result, these authors omitted this taxon from their analysis, a procedure that has been followed in the present contribution.

*Mourasuchus* is recovered as a monophyletic group, as in the other analyses that included all the species of the genus (Bona, Riff & Gasparini, 2013; Salas-Gismondi et al., 2015). Synapomorphies of this clade that are made of features uniquely found in *Mourasuchus* include: dentary symphysis very short, extending to the level of the first alveolous (Character 49-3); orbits smaller than infratemporal fenestrae (Character 181-2) and the prefrontal and frontal thickened, forming a marked knob at the anteromedial margin of the orbits (Character 182-1). Other shared characters of *Mourasuchus*, but that are not exclusive to the group, include: dentary linear between fourth and tenth alveoli (Character 50-2; shared with *Culebrasuchus*, *Tomistoma schlegelli* (Müller, 1838) *Thecachampsa americana* (Sellards, 1915), *Iharkutosuchus* and Gavialoidea); posterior teeth and alveoli of maxilla and/or dentary laterally compressed (Character 79-1; shared with *Paleosuchus*, *Kuttanacaiman*, *Procaimanoidea*, *Arambourgia* and *Osteolaemus*); nasals excluded, at least externally, from naris; nasals and premaxillae still in contact (Character 82-2; shared with *Globidentosuchus*, *Diplocynodon*, *Mecistops*, *T. schlegelli*, *T. americana*, *Boverisuchus magnifrons* Kuhn, 1938, *Borealosuchus*, *Eothoracosaurus*, *Eosuchus minor* (Marsh, 1870), *Eogavialis africanum* (Andrews, 1901) and *Gryposuchus colombianus* Langston, 1965); dorsal premaxillary processes long, extending beyond third maxillary alveolus (Character 90-1; shared with *Caiman brevirostris*, *Brachychampsa montana* Gilmore, 1911, *B. sealeyi* Williamson, 1996, *Albertochampsa*, *Stangerochampsa*, *Australosuchus*, *Kambara implexidens* Salisbury & Willis, 1996, *T. schlegelli*, *T. americana*, *Kentisuchus spenceri* Buckland, 1836, *Crocodylus acer* Cope, 1882, *Planocrania datagensiss* Li, 1976, *Iharkutosuchus* and Gavialoidea); frontoparietal suture linear between supratemporal fenestrae (Character 150-1; shared with *Kuttanacaiman*, *Tsoabichi*, *Paleosuchus*, *Melanosuchus niger*,* Caiman latirostris*, *Alligator mefferdi* Mook, 1946, *A. mississippiensis*, *A. thomsoni* Mook, 1923 *Arambourgia*, all *Diplocynodon* included in the analysis except *D. ratelii* Pomel, 1847, *Osteolaemus*, * Trilophosuchus*, *T. schlegelli*, *Asiatosuchus germanicus* Berg, 1966, all *Borealosuchus* included in the analysis except *B. sternbergii* (Gilmore, 1910), *Gavialis gangeticus*, *Gryposuchus colombianus*, Hylaochampsinae and *Bernissartia fagesii*).

The phylogenetic hypothesis obtained for *Mourasuchus* further splits this genus into a clade formed by *M. atopus* and *M. arendsi* and another, polytomous clade formed by *M. amazonensis*, UFAC-1424 and the new species *M. pattersoni*. *M. atopus* and *M. arendsi* are united by the presence of a premaxillary surface surrounded by a dorsoventrally developed rim (Character 86-2; Figs. 5A, 5C and 6; see S.1. in Supplemental Information 1, for further information), a morphology that is unique to these two taxa. Other features that differentiate *M. arendsi* and *M. atopus* from the other *Mourasuchus* species are the presence of a circular external naris (Character 83-0; Figs. 5 and 6) and a jugal lateromedially slender and dorsoventrally low (Character 187-0; Fig. 8; see S.1. in Supplemental Information 1 for further details).

The polytomy may be explained by the incomplete preservation of UFAC-1424 (see Bocquentin & Souza-Filho, 1990 and Bona, Degrange & Fernández, 2013). UFAC-1424 and *Mourasuchus amazonensis* share a lateromedially wide and dorsoventrally low jugal (Character 187-1; Figs. 8B and 8E). As such, it is possible that these two forms are more closely related to each other than to *M. pattersoni*, which exhibits an autapomorphy of a lateromedially wide and dorsoventrally high jugal (Character 187-2; Figs. 8C and 8F). *Mourasuchus pattersoni* (Figs. 5C and 6B) and *M. amazonensis* (Price, 1964) share the presence of an external naris wider than long (Character 83-1), but UFAC-1424 does not preserve the external naris (see Bona, Degrange & Fernández, 2013). *Mourasuchus pattersoni* also differs from *M. amazonensis* for having a circular incisive foramen, whilst this structure in the latter has a tri-lobed shape (Fig. 7). The incisive foramen is not preserved in *M. atopus* or in UFAC-1424; Bocquentin-Villanueva (1984) illustrates the incisive foramen of *M. arendsi* as having the anterior end significantly wider that the posterior one, thus assuming a roughly “reversed teardrop” shape. As the morphology of this structure could not be assessed directly in the holotype, we consider the interpretation as tentative for the time being. However, its eventual confirmation would not change significantly the phylogeny or the taxonomy of *Mourasuchus* in this paper, as it would represent only an autapomorphy of *M. arendsi*.

## Discussion

### The implications of the phylogenetic results for Caimaninae temporal distribution, paleoecology and taxonomy

The topology obtained in the phylogenetic analysis of this paper (Fig. 13) shows the presence of three Miocene taxa (*Culebrasuchus*, *Gnatusuchus* and *Globidentosuchus*) as the basalmost caimanines, while Paleocene and Eocene taxa such as *Eocaiman* and *Tsoabichi* appear as more derived. This suggests the presence of a ghost-lineage of this clade dating back from the Late Cretaceous or the Paleocene, the periods on which Caimaninae is thought to have been originated (Brochu, 2011). The presence of *Kuttanacaiman* as the sister-taxon of the clade formed by *Tsoabichi* and *Paleosuchus* also suggests a significant ghost lineage dating back from at least the Eocene. Future phylogenetic analyses and collection efforts in Paleogene deposits throughout the Americas are needed for a better understanding on the phylogeny and temporal distribution of the Caimaninae taxa.

Following Salas-Gismondi et al. (2015) in considering *Gnatusuchus*, *Globidentosuchus*, *Kuttanacaiman* and *Caiman wannlangstoni* as predominantly durophagous taxa, this topology indicates that the durophagous habit arose at least twice within Caimaninae. This perspective of independently origins of durophagy is in concordance with the morphological plasticity present in the extant caimanines *Caiman crocodilus* and *C. latirostris*, in which some populations or individuals have been found to present adaptations to a more durophagous diet (Langston, 1965; Ösi & Barrett, 2011). Distinctly, other, more complex ecological adaptations such as that of large-bodied top predator (*Purussaurus*) and gulp-feeding predator (*Mourasuchus*, see below) have been invariably found to have arisen only once (e.g., Bona, Riff & Gasparini, 2013; Salas-Gismondi et al., 2015; Fig. 13).

The present analysis recovers *Melanosuchus niger* in a derived position among the Caiman clade, a topology already recovered in most previous morphological analyses (e.g., Brochu, 1999; Brochu, 2010; Brochu, 2011; Aguilera, Riff & Bocquentin-Villanueva, 2006; Bona, 2007; Hastings et al., 2013; Scheyer et al., 2013; Hastings, Reisser & Scheyer, 2016). This suggests that *Melanosuchus* could actually be considered as belonging to *Caiman*, as already proposed by previous studies (e.g., Poe, 1997). Furthermore, *Caiman wannlangstoni* was recovered in this analysis outside the Jacarea clade, in which all the other *Caiman* species are present (Fig. 13). This suggests that *C. wannlangstoni* may also not belong to *Caiman*. However, taxonomic revisions of these taxa are out of the scope of the present contribution and should be addressed in future studies.

### The crocodylian diversity in the Urumaco Formation

The Urumaco Formation has a large and unique assemblage of fossil crocodylians (see Aguilera, 2004; Sánchez-Villagra & Aguilera, 2006 and Riff et al., 2010 for reviews). In particular for *Mourasuchus*, this geologic unit already had occurrences of the species *M. arendsi* (Bocquentin-Villanueva, 1984; Scheyer & Delfino, 2016). The description of the new taxon *Mourasuchus pattersoni* sp. nov. in this paper makes the diversity of the Urumaco Formation equal to the one of the Solimões Formation, for which there are also two species described: *M. amazonensis* and *M. arendsi* (Souza-Filho & Guilherme, 2011b). With *M. pattersoni* sp. nov., the caimanine diversity in the Urumaco Formation reaches a total of seven species, which also includes *Globidentosuchus brachyrostris* Scheyer et al., 2013, *Melanosuchus fisheri*, *Purussaurus mirandai*, *Caiman brevirostris* and *Caiman latirostris* (Aguilera, 2004; Riff et al., 2010; Scheyer et al., 2013)—even though this latter occurrence was originally assigned as *Caiman lutescens* (Sánchez-Villagra & Aguilera, 2006; Scheyer & Moreno-Bernal, 2010).

The caimanine diversity for the Urumaco Formation by itself shows the existence of four different ecological niches to be performed by crocodyliforms: medium-sized generalist predator (*Caiman*, *Melansosuchus*); giant-sized top predator (*Purussaurus*, see Aureliano et al., 2015); durophagous predator (*Globidentosuchus*, see Salas-Gismondi et al., 2015) and gulp-feeding (*Mourasuchus*, see below). The existence of longirostrine, predominantly piscivorous taxa such as gavialoids (*Gryposuchus*, *Hesperogavialis*, *Ikanogavialis*) and crocodyloids (*Thecachampsa*, *Charactosuchus*) add a fifth ecological niche performed by crocodylians to the paleofauna of the Urumaco Formation. Thus all but one of the ecological niches performed by crocodyliforms in the Miocene of South America are present in the Urumaco Formation: the only absence is that of the terrestrial predatory role performed by the sebecids, a group whose last record dates back to the middle Miocene (Langston, 1965; Salas-Gismondi et al., 2007; Paolillo & Linares, 2007; Kellner, Pinheiro & Campos, 2014).

The taxonomic and ecomorphological diversity of crocodyliforms in the Urumaco Formation is shared by other Miocene units throughout South America. Among these, there are the middle Miocene Honda Group of Colombia (Langston, 1965; Langston & Gasparini, 1997), the Fitzcarrald Arch (Salas-Gismondi et al., 2007) and the Pebas Formation of Peru (Salas-Gismondi et al., 2015), and the late Miocene Solimões (Riff et al., 2010) and Ituzaingó (Bona, Riff & Gasparini, 2013) formations in Brazil and Argentina, respectively. The reasons for the presence of such remarkable diversities in the Miocene of South America, especially in the region of the present-day Amazon rainforest, have been discussed by several authors (e.g., Cozzuol, 2006; Latrubesse et al., 2010; Riff et al., 2010; Scheyer & Moreno-Bernal, 2010; Scheyer et al., 2013; Souza et al., 2016). Among these, the most frequently cited are the high temperatures and humidity of the region (for example see Head et al., 2009, for temperature estimates of equatorial South America during the Paleogene), which are suitable to crocodylian physiology (Brochu, 2011) and the heterogeneity of environments and habitats, which allowed the existence of diversity in several environmental factors but especially in that of prey items that permitted the evolution of an array of distinct feeding habits (Latrubesse et al., 2010).

Another important factor is the time, namely the long-term environmental stability that equatorial areas are likely to have across geological time, which allied with the abundance of primary productivity of such areas allowed the evolution of several distinct biological lineages throughout its history (Darwin, 1859; Wallace, 1878; Fischer, 1960; Mittelbach et al., 2007). This scenario is frequently evoked to explain the existence of a larger extant biodiversity in the equatorial and tropical areas when compared with the temperate ones. The environmental stability of those areas would allow not only the origin and evolution of new taxa, but would also allow these to endure for a much longer time in their original habitats than in temperate areas (Jablonski, Roy & Valentine, 2006; McKenna & Farrell, 2006; Mittelbach et al., 2007). This scenario may be related to the topology obtained in our phylogenetic analysis, which shows basal taxa of Caimaninae (*Culebrasuchus*, *Gnatusuchus* and *Globidentosuchus*) occurring in the Miocene, together with more derived taxa such as *Purussaurus* and *Mourasuchus* (Fig. 13). The persistence of these basal taxa through to the Miocene may be related to an environmental stability of the equatorial region of South America during the Paleogene and Neogene (e.g., Souza et al., 2016).

### The feeding habits and paleoecology of *Mourasuchus*

The unusual anatomy of *Mourasuchus* has provoked a debate about the feeding habits of the group, which have never been properly defined (see Langston, 1965; Langston, 2008; Riff et al., 2010; Bona, Degrange & Fernández, 2013; Tineo et al., 2014). Several anatomical characteristics of *Mourasuchus*, such as a long, wide, dorsoventrally flattened rostrum (Figs. 2 and 3), slender mandibles with a short symphysis (Fig. 4), small teeth and short cervical vertebrae indicate that *Mourasuchus* was unlikely to be able to capture, hold or ingest large prey (see Langston, 1965; Langston, 2008; Bocquentin-Villanueva, 1984; Tineo et al., 2014) in the way of many current crocodylians (e.g., Busbey, 1994). It has been suggested that *Mourasuchus* could feed preferably on small animals, namely crustaceans and small fish (Langston, 1965).

In this paper, we follow Langston (1965) in suggesting that *Mourasuchus* fed preferably on small-sized invertebrate animals, mainly mollusks (bivalves and gastropods), crustaceans (crabs and shrimps) and small fish. However, here it is further suggested that *Mourasuchus*’ feeding behavior consisted mainly in the use of the large area of the snout to collect a large number of individuals of the prey items at the same time, with the ventral portion of the rostrum. This perspective would explain the long, wide, dorsoventrally flattened rostrum of this group, which would be suitable to collect large amounts of small prey than to capture, hold or dismember large, vertebrate animals. In relation to the rostral morphology of *Mourasuchus*, Walmsley et al. (2013) state that the crocodylian skull exhibited a “trade-off” along its evolutionary history between a long, slender rostrum that provided speediness, and a shorter, yet more robust rostrum that provides strength in biting and consequently in the capture of prey. The “platyrostral-broad” morphology of *Mourasuchus* presents a case in which the rostrum does not provide either speediness or strength to the bite. Instead, an increase in area, to optimize the preferential capture of a large amount of small prey, seems to be the evolutive advantage that this rostral morphology provided to the individuals of *Mourasuchus*.

In this context, it has been suggested that the ventral portion of the rostrum would function as a “fishing net” or “gular sac” that could reminisce those of the pelicans (Langston, 1965). The presence of such structure with a similar function had already been theorized by Nopcsa (1926) for *Stomatosuchus inermis* Stromer, 1925, a crocodyliform from the Late Cretaceous of Egypt whose skull anatomy resembles that of *Mourasuchus* (Sereno & Larsson, 2009). However, whether *Mourasuchus* actually had such structure is currently not known.

Langston (1965) suggested that the feeding behavior of *Mourasuchus* could be named as a “straining technique”, which later authors have named it as “filter-feeding” (e.g., Riff et al., 2010; Bona, Degrange & Fernández, 2013). The small animals upon which *Mourasuchus* preferably fed are most frequently found either within a body of water or in the middle of a substratum, either biotic (plants) or abiotic (mud or sand). As such, *Mourasuchus* would probably frequently capture both the edible and the non-edible material in its mouth at the same time. Thus, the presence of a “filtering” technique would be beneficial if it could separate the edible from the non-edible before swallowing. However, there is still no evidence to support that *Mourasuchus* could perform a filtering behavior or how it could be performed. As such, we suggest the term “gulp-feeding” instead of “filter-feeding” to describe the feeding habits of *Mourasuchus* proposed in this paper.

These hypotheses on *Mourasuchus* feeding ecology must, however, be thoroughly assessed in further research, especially one of biomechanics, which have not been performed for this genus yet (Bona, Degrange & Fernández, 2013).

## Conclusions

Previously regarded as a “probable” specimen of *Mourasuchus arendsi* by Langston (2008), this thorough study of the specimen MCNC-PAL-110-72V recognizes it as a new *Mourasuchus* species, named as *M. pattersoni* sp. nov. This new taxon is the fourth recorded for *Mourasuchus*, and the second of this genus recorded for the Urumaco Formation.

The phylogenetic analysis of Caimaninae performed in this paper shows some new perspectives about the evolution of the group, such as that the newly described forms *Culebrasuchus*, *Gnatusuchus* and *Globidentosuchus* form successive sister-taxa to the remaining caimanines, and the recovery of *Kuttanacaiman* and *Caiman wannlagstoni* in different positions from those of their original phylogenetic assessments (Salas-Gismondi et al., 2015). Other results obtained are generally congruent with previous analyses, such as the monophyly of *Eocaiman*, the position of *Tsoabichi* as close to *Paleosuchus* and the recovery of *Mourasuchus* as a sister taxon of *Purussaurus*.

Additionally, this contribution discusses the feeding habits of *Mourasuchus*, defending that this taxon fed preferably on small animals, especially invertebrates (crustaceans and mollusks). It also suggests that the unusual skull morphology of this group evolved probably to provide a larger covering of area by the rostrum. This would allow a greater efficiency in the capture of large amounts of the small animals that constituted the majority of the prey of *Mourasuchus*. It is also considered that, even though it is possible that *Mourasuchus* performed some sort of selection of the edible matter from non-edible matter in the mouth that could be called “filtering”, it is not possible yet to confirm if such a procedure was performed by this taxon. As such, it is considered that “gulp-feeding” is a more appropriate term than “filter-feeding” to describe the feeding behavior of *Mourasuchus*.

The fedding habits proposed in this paper for *Mourasuchus* are, however, still hypotheses. Further studies and the finding of more complete specimens are required for these to be thoroughly tested.

##  Supplemental Information

10.7717/peerj.3056/supp-1 Supplemental Information 1 Supplemental Information Characters, scorings and consulted sources (collection specimens and bibliography) used in the phylogenetic analysis.

10.7717/peerj.3056/supp-2 Supplemental Information 2 Phylogenetic Matrix Matrix used for the phylogenetic analysis in this study (nexus format).

10.7717/peerj.3056/supp-3 Figure S1 The topology of Eusuchia from the strict consensus of 120 cladograms (629 steps; ensemble CI = 0.391; ensemble RI = 0.810) obtained by the phylogenetic analysis performed in this paper

## Institutional abbreviations

AMNH: American Museum of Natural History, New York, United States
CIAAP: Centro de Investigaciones Antropológicas, Arqueológicas e Paleontógicas, Universidad Nacional Experimental Francisco de Miranda (UNEFM), Coro, Venezuela
DGM: Divisão de Geologia e Mineralogia, Serviço Geológico do Brasil (CPRM), Rio de Janeiro, Brazil
MACN: Museo Argentino de Ciéncias Naturales “Bernardino Rivadávia”, Buenos Aires, Argentina
MCNC: Museo de Ciéncias Naturales de Caracas, Caracas, Venezuela
MCZ: Museum of Comparative Zoology, Boston, United States
MLP: Museo de La Plata, La Plata, Argentina
UCMP: University of California Museum of Paleontology, Berkeley, United States
UFAC: Universidade Federal do Acre, Rio Branco, Brazil.

## Additional Information and Declarations

### Data Availability

The following information was supplied regarding data availability: The raw data has been supplied as Supplementary File.

### New Species Registration

The following information was supplied regarding the registration of a newly described species: *Mourasuchus*: urn:lsid:zoobank.org:act:CDECD729-E9F5-4DD3-8185-87BDE8380E87 *Mourasuchus pattersoni*: urn:lsid:zoobank.org:act:A4DC0A3D-3F38-4E8C-89EF-AF53CDD69A46 Publication LSID: urn:lsid:zoobank.org:pub:D2F1F0A2-5E2B-4111-A6E4-AC78480CF3B2.
