# Supplementary material for: A new Mourasuchus (Alligatoroidea, Caimaninae) from the late Miocene of Venezuela, the phylogeny of Caimaninae and considerations on the feeding habits of Mourasuchus
Source: PeerJ. 2017 Mar 7;5:e3056. doi: 10.7717/peerj.3056 (PMC5344020; doi:10.7717/peerj.3056)
Supplement: Supplemental Information 1 — Characters, scorings and consulted sources (collection specimens and bibliography) used in the phylogenetic analysis. [file peerj-05-3056-s001.docx]

**Supplemental Information**

**A new *Mourasuchus* (Alligatoroidea, Caimaninae) from the Late Miocene of Venezuela, the phylogeny of Caimaninae and considerations on the feeding habits of *Mourasuchus***

Giovanne M. Cidade^1^, Andrés Solórzano^2^, Ascanio Daniel Rincón^2^, Douglas Riff^3^ & Annie Schmaltz Hsiou^1^

^1^Departamento de Biologia, Faculdade de Filosofia, Ciências e Letras de Ribeirão Preto, Universidade de São Paulo, Ribeirão Preto, São Paulo, Brazil

^2^Centro de Ecología, Instituto Venezolano de Investigaciones Científicas, San António de los Altos, Miranda, Venezuela

^3^Instituto de Biologia, Universidade Federal de Uberlândia, Uberlândia, Minas Gerais, Brazil

Corresponding author: Giovanne Cidade; giovannecidade@hotmail.com

**List of institutional abbreviations**

**AMNH**: American Museum of Natural History, New York, United States;

**CIAAP**: Centro de Investigaciones Antropológicas, Arqueológicas e Paleontógicas, Universidad Nacional Experimental Francisco de Miranda (UNEFM), Coro, Venezuela;

**DGM***: Museu de Ciências da Terra, Serviço Geológico do Brasil (CPRM), Rio de Janeiro (RJ), Brazil;

**HLMD-Me**: Hessisches Landmuseum, Darmstadt, Germany;

**MCNC**: Museo de Ciéncias Naturales de Caracas, Caracas, Venezuela;

**MCT***: Museu de Ciências da Terra, Serviço Geológico do Brasil (CPRM), Rio de Janeiro (RJ), Brazil;

**MLP**: Museo de La Plata, La Plata, Argentina;

**MNHN**: Muséum National d'Histoire Naturelle, Paris, France;

**MPEF-PV**: Museu Paleontológico Egídio Feruglio, Trelew, Argentina;

**SMM**: Science Museum of Minnesota, Saint Paul, United States;

**SMNS**: Staatliches Museum fur Naturkunde, Stuttgart, Germany;

**UCMP**: University of California Museum of Paleontology, Berkeley, United States;

**UFAC**: Universidade Federal do Acre, Rio Branco, Brazil;

**YPM HER R**: Peabody Museum of Natural History, Herpetology, Yale University, New Haven, United States.

* Both DGM and MCT refer to the same institution, but as different works use each of these acronyms for registration numbers, it was decided to keep the acronym used by each bibliographical reference, hence the listing of two acronyms for the same institution.

**S.1. List of characters used in the phylogenetic analysis**

All characters taken from Brochu (2011), unless otherwise noted. Characters from 1 to 106 have the same number as in Brochu (2011); characters 107 to 180 are subtracted by one from the numbering used by Brochu (2011) due to the exclusion of its character “107” from the present analysis. Some of the rephrased characters, and the new characters, are followed by pertinent comments.

1. Ventral tubercle of proatlas more than one half (0) or no more than one half (1) the width of the dorsal crest

2. Fused proatlas boomerang-shaped (0), strapshaped (1), or massive and block-shaped (2)

3. Proatlas with prominent anterior process (0) or lacks anterior process (1)

4. Proatlas has tall dorsal keel (0) or lacks tall dorsal keel; dorsal side smooth (1)

5. Atlas intercentrum wedge-shaped in lateral view, with insignificant parapophyseal processes (0), or plate-shaped in lateral view, with prominent parapophyseal processes at maturity (1)

6. Dorsal margin of atlantal rib generally smooth with modest dorsal process (0) or with prominent process (1)

7. Atlantal ribs without (0) or with (1) very thin medial laminae at anterior end

8. Atlantal ribs lack (0) or possess (1) large articular facets at anterior ends for each other

9. Axial rib tuberculum wide, with broad dorsal tip (0) or narrow, with acute dorsal tip (1)

10. Axial rib tuberculum contacts diapophysis late in ontogeny, if at all (0) or early in ontogeny (1)

11. Anterior half of axis neural spine orientated horizontally (0) or slopes anteriorly (1)

12. Axis neural spine crested (0) or not crested (1)

13. Posterior half of axis neural spine wide (0) or narrow (1)

14. Axis neural arch lacks (0) or possesses (1) a lateral process (diapophysis)

15. Axial hypapophysis located toward the centre of centrum (0) or toward the anterior end of centrum (1)

16. Axial hypapophysis without (0) or with (1) deep fork

17. Hypapophyseal keels present on 11th vertebra behind atlas (0), 12th vertebra behind atlas (1), or tenth vertebra behind atlas (2)

18. Third cervical vertebra (first postaxial) with prominent hypapophysis (0) or lacks prominent hypapophysis (1)

19. Neural spine on third cervical long, dorsal tip at least half the length of the centrum without the cotyle (0) or short, dorsal tip acute and less than half the length of the centrum without the cotyle (1)

20. Cervical and anterior dorsal centra lack (0) or bear (1) deep pits on the ventral surface of the centrum

21. Presacral centra amphicoelous (0) or procoelous (1)

22. Anterior sacral rib capitulum projects far anteriorly of tuberculum and is broadly visible in dorsal view (0), or anterior margins of tuberculum and capitulum nearly in same plane, and capitulum largely obscured dorsally (1)

23. Scapular blade flares dorsally at maturity (0) or sides of scapular blade subparallel; minimal dorsal flare at maturity (1)

24. Deltoid crest of scapula very thin at maturity, with sharp margin (0) or very wide at maturity, with broad margin (1)

25. Scapulocoracoid synchondrosis closes very late in ontogeny (0) or relatively early in ontogeny (1)

26. Scapulocoracoid facet anterior to glenoid fossa uniformly narrow (0) or broad immediately anterior to glenoid fossa, and tapering anteriorly (1)

27. Proximal edge of deltopectoral crest emerges smoothly from proximal end of humerus and is not obviously concave (0) or emerges abruptly from proximal end of humerus and is obviously concave (1)

28. M. teres major and M. dorsalis scapulae insert separately on humerus; scars can be distinguished dorsal to deltopectoral crest (0) or insert with common tendon; single insertion scar (1)

29. Olecranon process of ulna narrow and subangular (0) or wide and rounded (1)

30. Distal extremity of ulna expanded transversely with respect to long axis of bone; maximum width equivalent to that of proximal extremity (0) or proximal extremity considerably wider than distal extremity (1)

31. Interclavicle flat along length, without dorsoventral flexure (0) or with moderate dorsoventral flexure (1) or with severe dorsoventral flexure (2)

32. Anterior end of interclavicle flat (0) or rod-like (1)

33. Iliac anterior process prominent (0) or virtually absent (1)

34. Dorsal margin of iliac blade rounded with smooth border (0) or rounded, with modest dorsal indentation (1) or rounded, with strong dorsal indentation (wasp-waisted; 2) or narrow, with dorsal indentation (3) or rounded with smooth border; posterior tip of blade very deep (4)

35. Supraacetabular crest narrow (0) or broad (1)

36. Limb bones relatively robust, and hindlimb much longer than forelimb at maturity (0) or limb bones very long and slender (1)

37. M. caudofemoralis with single head (0) or with double head (1)

38. Dorsal osteoderms not keeled (0) or keeled (1)

39. Dorsal midline osteoderms rectangular (0) or nearly square (1)

40. Four (0), six (1), eight (2), or ten (3) contiguous dorsal osteoderms per row at maturity

41. Nuchal shield grades continuously into dorsal shield (0) or differentiated from dorsal shield; four nuchal osteoderms (1) or differentiated from dorsal shield; six nuchal osteoderms with four central and two lateral (2) or differentiated from dorsal shield; eight nuchal osteoderms in two parallel rows (3)

42. Ventral armour absent (0) or single ventral osteoderms (1) or paired ventral ossifications that suture together (2)

43. Anterior margin of dorsal midline osteoderms with anterior process (0) or smooth, without process (1)

44. Ventral scales have (0) or lack (1) follicle gland pores

45. Ventral collar scales not enlarged relative to other ventral scales (0) or in a single enlarged row (1) or in two parallel enlarged rows (2)

46. Median pelvic keel scales form two parallel rows along most of tail length (0) or form single row along tail (1) or merge with lateral keel scales (2)

47. Alveoli for dentary teeth 3 and 4 nearly same size and confluent (0), or fourth alveolus larger than third, and alveoli are separated (1), or 3 and 4 are nearly the same size and separated (2). From Salas-Gimsondi *et al*. (2015), character 47, modified from Brochu (2011), character 47.

48. Anterior dentary teeth strongly procumbent (0) or project anterodorsally (1)

49. Dentary symphysis extends to fourth or fifth alveolus (0) or sixth to eighth alveolus (1) or behind eighth alveolus (2) **or symphysis very short, extending to the level of the first alveolous (3).** Rephrased.

The state “3” represents the morphology seen in *Mourasuchus*, where the mandibular symphysis is very short, with its length extending only roughly to the level of the first alveolus (see Langston, 1965; Bocquentin & Souza-Filho, 1990). This feature, however, could not be scored for *M. pattersoni* sp. nov., as the plaster cover the symphysial area precludes an exact assessment of this character.

50. Dentary gently curved (0), deeply curved (1), or linear (2) between fourth and tenth alveoli

51. Largest dentary alveolus immediately caudal to fourth is (0) 13 or 14, (1) 13 or 14 and a series behind it, (2) 11 or 12, or (3) no differentiation, or (4) behind 14

52. Splenial with anterior perforation for mandibular ramus of cranial nerve V (0) or lacks anterior perforation for mandibular ramus of cranial nerve V (1)

53. Mandibular ramus of cranial nerve V exits splenial anteriorly only (0) or splenial has singular perforation for mandibular ramus of cranial nerve V posteriorly (1) or splenial has double perforation for mandibular ramus of cranial nerve V posteriorly (2)

54. Splenial participates in mandibular symphysis; splenial symphysis adjacent to no more than five dentary alveoli (0) or splenial excluded from mandibular symphysis; anterior tip of splenial passes ventral to Meckelian groove (1) or splenial excluded from mandibular symphysis; anterior tip of splenial passes dorsal to Meckelian groove (2) or deep splenial symphysis, longer than five dentary alveoli; splenial forms wide V within symphysis (3) or deep splenial symphysis, longer than five dentary alveoli; splenial constricted within symphysis and forms narrow V (4)

55. Coronoid bounds posterior half of foramen intermandibularis medius (0) or completely surrounds foramen intermandibularis medius at maturity (1) or obliterates foramen intermandibularis medius at maturity (2)

56. Superior edge of coronoid slopes strongly anteriorly (0) or almost horizontal (1)

57. Inferior process of coronoid laps strongly over inner surface of Meckelian fossa (0) or remains largely on medial surface of mandible (1)

58. Coronoid imperforate (0) or with perforation posterior to foramen intermandibularis medius (1)

59. Process of splenial separates angular and coronoid (0) or no splenial process between angular and coronoid (1)

60. Angular–surangular suture contacts external mandibular fenestra at posterior angle at maturity (0) or passes broadly along ventral margin of external mandibular fenestra late in ontogeny (1)

61. Anterior processes of surangular unequal (0) or subequal to equal (1)

62. Surangular with spur bordering the dentary tooth row lingually for at least one alveolus length (0) or lacking such spur (1)

63. External mandibular fenestra absent (0) or present (1) or present and very large; most of foramen intermandibularis caudalis visible in lateral view (2)

64. Surangular–dentary suture intersects external mandibular fenestra anterior to posterodorsal corner (0) or at posterodorsal corner (1)

65. Angular extends dorsally toward or beyond anterior end of foramen intermandibularis caudalis; anterior tip acute (0) or does not extend dorsally beyond anterior end of foramen intermandibularis caudalis; anterior tip very blunt (1)

66. Surangular–angular suture lingually meets articular at ventral tip (0) or dorsal to tip (1)

67. Surangular continues to dorsal tip of lateral wall of glenoid fossa (0) or truncated and not continuing dorsally (1)

68. Articular–surangular suture simple (0) or articular bears anterior lamina dorsal to lingual foramen (1) or articular bears anterior lamina ventral to lingual foramen (2) or bears laminae above and below foramen (3)

69. Lingual foramen for articular artery and alveolar nerve perforates surangular entirely (0) or perforates surangular/angular suture (1)

70. Foramen aereum at extreme lingual margin of retroarticular process (0) or set in from margin of retroarticular process (1)

71. Retroarticular process projects posteriorly (0) or projects posterodorsally (1)

72. Surangular extends to posterior end of retroarticular process (0) or pinched off anterior to tip of retroarticular process (1)

73. Surangular–articular suture orientated anteroposteriorly (0) or bowed strongly laterally (1) within glenoid fossa

74. Sulcus between articular and surangular (0) or articular flush against surangular (1)

75. Dorsal projection of hyoid cornu flat (0) or rodlike (1)

76. Dorsal projection of hyoid cornu narrow, with parallel sides (0) or flared (1)

77. Lingual osmoregulatory pores small (0) or large (1)

78. Tongue with (0) or without (1) keratinized surface

79. Teeth and alveoli of maxilla and/or dentary circular in cross-section (0), or posterior teeth laterally compressed (1), or all teeth compressed (2)

80. Maxillary and dentary teeth with smooth carinae (0), or serrated (1), or with neither carinae nor serrations (2). From Salas-Gismondi *et al*. (2015), character 80, modified from Brochu (2011), character 80.

81. Naris projects anterodorsally (0) or dorsally (1)

82. External naris bisected by nasals (0) or nasals contact external naris, but do not bisect it (1) or nasals excluded, at least externally, from naris; nasals and premaxillae still in contact (2) or nasals and premaxillae not in contact (3)

83. Naris circular or keyhole-shaped (0) or wider than long (1) **or longer than wide (2)** or anteroposteriorly long and prominently teardrop-shaped (3). Rephrased.

The new state separates the morphology seen in the *Purussaurus* species, in which the external naris is longer than wide. This morphology is seen in *P. neivensis* (see Langston, 1965, Fig. 37) *P. mirandai* (see Aguilera *et al*., 2006, Figs. 2, 3 and 7) and *P. brasiliensis* (see Aguilera *et al*., 2006, Fig. 5) even though the latter was not included in the analysis.

84. External naris of reproductively mature males (0) remains similar to that of females or (1) develops bony excrescence (ghara)

85. External naris (0) opens flush with dorsal surface of premaxillae or (1) circumscribed by thin crest

86. Premaxillary surface lateral to naris smooth (0) or with deep notch lateral to naris (1) **or surrounded by a dorsoventrally developed rim (2).** Rephrased.

The new state (2) described the morphology found in *Mourasuchus arendsi* (Fig. 5-B and 6-A) and possibly in *M. atopus* (Fig. 5-A), in which there is an accentuated, dorsoventrally developed rim surrounding the external naris in the dorsal surface of the premaxilla. This structure can be fully seen in *M. arendsi*, but not in *M. atopus* as this species only preserves part of the right premaxilla; nevertheless, the elevation surrounding the opening of the external naris in this taxon is here considered as representing the same structure seen in *M. arendsi*.

The other species of *Mourasuchus*, *M. amazonensis* and *M. pattersoni* sp. nov. (Fig. 5-C and 6-B) do not show this rim, although this absence must be seen with caution regarding *M. amazonensis* as the skull of the holotype cannot be seen at the moment, and the only source of information regarding the anatomy of this skull in dorsal view is the drawing offered by Price (1964), which shows premaxillae with a large, wider than long external naris but without any reference to the presence of a rim around it, similarly to *M. pattersoni* sp. nov.

This character was, originally, created to describe the existence of a “notch” in the lateral margins of the external naris in *Alligator* (Brochu, 1997). This notch is very different from the rim seen in *Mourasuchus arendsi* and *M. atopus*, since the first is only a slight elevation of the nasal margins, and the second is much more accentuated and uneven relative to the adjacent lateral premaxillary surface. Nevertheless, the two features may be accommodated in the same character as they are structures that occupy practically the same area (the lateral margin of the external naris) and would be thus extremely unlikely that the two features could occur simultaneously in one taxon; this perspective allows these features to be considered independent from each other.

87. Premaxilla has five teeth (0) or four teeth (1) early in posthatching ontogeny

88. Incisive foramen small, less than half the greatest width of premaxillae (0) or large, more than half the greatest width of premaxillae (1) or large, and intersects premaxillary–maxillary suture (2)

89. Incisive foramen completely situated far from premaxillary tooth row, at the level of the second or third alveolus (0) or abuts premaxillary tooth row (1) or projects between first premaxillary teeth (2)

90. Dorsal premaxillary processes short, not extending beyond third maxillary alveolus (0) or long, extending beyond third maxillary alveolus (1)

91. Dentary tooth 4 occludes in notch between premaxilla and maxilla early in ontogeny (0) or occludes in a pit between premaxilla and maxilla; no notch early in ontogeny (1)

92. All dentary teeth occlude lingual to maxillary teeth (0) or occlusion pit between seventh and eighth maxillary teeth; all other dentary teeth occlude lingually (1) or dentary teeth occlude in line with maxillary tooth row (2)

93. Largest maxillary alveolus is no. 3 (0), no. 5 (1), no. 4 (2), nos. 4 and 5 are same size (3), no. 6 (4), or maxillary teeth homodont (5), or maxillary alveoli gradually increase in diameter posteriorly toward penultimate alveolus (6)

94. Maxillary tooth row curved medially or linear (0) or curves laterally broadly (1) posterior to first six maxillary alveoli

95. Dorsal surface of rostrum curves smoothly (0) or bears medial dorsal boss (1)

96. Canthi rostralii absent or very modest (0) or very prominent (1) at maturity

97. Preorbital ridges absent or very modest (0) or very prominent (1) at maturity

98. Antorbital fenestra present (0) or absent (1)

99. Vomer entirely obscured by premaxilla and maxilla (0) or exposed on palate at premaxillary–maxillary suture (1)

100. Vomer entirely obscured by maxillae and palatines (0) or exposed on palate between palatines (1)

101. Surface of maxilla within narial canal imperforate (0) or with a linear array of pits (1)

102. Medial jugal foramen small (0) or very large (1)

103. Maxillary foramen for palatine ramus of cranial nerve V small or not present (0) or very large (1)

104. Ectopterygoid abuts maxillary tooth row (0) or maxilla broadly separates ectopterygoid from maxillary tooth row (1)

105. Maxilla terminates in palatal view anterior to lower temporal bar (0) or comprises part of the lower temporal bar (1)

106. Penultimate maxillary alveolus less than (0) or more than (1) twice the diameter of the last maxillary alveolus

107. Dorsal half of prefrontal pillar narrow (0) or expanded anteroposteriorly (1)

108. Medial process of prefrontal pillar expanded dorsoventrally (0) or anteroposteriorly (1)

109. Prefrontal pillar solid (0) or with large pneumatic recess (1)

110. Medial process of prefrontal pillar wide (0) or constricted (1) at base

111. Maxilla has linear medial margin adjacent to suborbital fenestra (0) or bears broad shelf extending into fenestra, making lateral margin concave (1)

112. Anterior face of palatine process rounded or pointed anteriorly (0) or notched anteriorly (1)

113. Anterior ectopterygoid process tapers to a point (0) or forked (1)

114. Palatine process extends (0) or does not extend (1) significantly beyond anterior end of suborbital fenestra

115. Palatine process generally broad anteriorly (0) or in form of thin wedge (1)

116. Lateral edges of palatines smooth anteriorly (0) or with lateral process projecting from palatines into suborbital fenestrae (1)

117. Palatine–pterygoid suture nearly at (0) or far from (1) posterior angle of suborbital fenestra

118. Pterygoid ramus of ectopterygoid straight, posterolateral margin of suborbital fenestra linear (0) or ramus bowed, posterolateral margin of fenestra concave (1)

119. Lateral edges of palatines parallel posteriorly (0) or flare posteriorly, producing shelf (1)

120. Anterior border of the choana is comprised of the palatines (0) or choana entirely surrounded by pterygoids (1)

121. Choana projects posteroventrally (0) or anteroventrally (1) at maturity

122. Pterygoid surface lateral and anterior to internal choana flush with choanal margin (0) or pushed inward anterolateral to choanal aperture (1) or pushed inward around choana to form neck surrounding aperture (2) or everted from flat surface to form neck surrounding aperture (3)

123. Posterior rim of internal choana not deeply notched (0) or deeply notched (1)

124. Internal choana not septate (0) or with septum that remains recessed within choana (1) or with septum that projects out of choana (2)

125. Ectopterygoid–pterygoid flexure disappears during ontogeny (0) or remains throughout ontogeny (1)

126. Ectopterygoid extends (0) or does not extend (1) to posterior tip of lateral pterygoid flange at maturity

127. Lacrimal makes broad contact with nasal; no posterior process of maxilla (0) or maxilla with posterior process within lacrimal (1) or maxilla with posterior process between lacrimal and prefrontal (2) or prefrontal extending an anterior process that separates the nasal from the lacrimal (3). From Aguilera *et al*. (2006), modified from Brochu (1999), character 93.

128. Prefrontals separated by frontals and nasals (0) or prefrontals meet medially (1)

129. Lacrimal longer than prefrontal (0), or prefrontal longer than lacrimal (1), or lacrimal and prefrontal both elongate and nearly the same length (2)

130. Anterior tip of frontal forms simple acute point (0), or forms broad, complex sutural contact either with the nasals or prefrontals (1). From Salas-Gimsondi *et al*. (2015), character 131, modified from Brochu (2011), character 131.

131. Ectopterygoid extends along medial face of postorbital bar (0) or stops abruptly ventral to postorbital bar (1)

132. Postorbital bar massive (0) or slender (1)

133. Postorbital bar bears process that is prominent, dorsoventrally broad, and divisible into two spines (0) or bears process that is short and generally not prominent (1)

134. Ventral margin of postorbital bar flush with lateral jugal surface (0) or inset from lateral jugal surface (1)

135. Postorbital bar continuous with anterolateral edge of skull table (0) or inset (1)

136. Margin of orbit flush with skull surface (0) or dorsal edges of orbits upturned (1) or orbital margin telescoped (2)

137. Ventral margin of orbit circular (0) or with prominent notch (1)

138. Palpebral forms from single ossification (0) or from multiple ossifications (1)

139. Quadratojugal spine prominent at maturity (0) or greatly reduced or absent at maturity (1)

140. Quadratojugal spine low, near posterior angle of infratemporal fenestra (0) or high, between posterior and superior angles of infratemporal fenestra (1)

141. Quadratojugal forms posterior angle of infratemporal fenestra (0) or jugal forms posterior angle of infratemporal fenestra (1) or quadratojugal–jugal suture lies at posterior angle of infratemporal fenestra (2)

142. Postorbital neither contacts quadrate nor quadratojugal medially (0) or contacts quadratojugal, but not quadrate, medially (1) or contacts quadrate and quadratojugal at dorsal angle of infratemporal fenestra (2) or contacts quadratojugal with significant descending process (3)

143. Quadratojugal bears long anterior process along lower temporal bar (0) or bears modest process, or none at all, along lower temporal bar (1)

144. Quadratojugal extends to superior angle of infratemporal fenestra (0) or does not extend to superior angle of infratemporal fenestra; quadrate participates in fenestra (1)

145. Postorbital–squamosal suture orientated ventrally (0) or passes medially (1) ventral to skull table

146. Dorsal and ventral rims of squamosal groove for external ear valve musculature parallel (0) or squamosal groove flares anteriorly (1)

147. Squamosal–quadrate suture extends dorsally along posterior margin of external auditory meatus (0) or extends only to posteroventral corner of external auditory meatus (1)

148. Posterior margin of otic aperture smooth (0) or bowed (1)

149. Frontoparietal suture deeply within supratemporal fenestra; frontal prevents broad contact between postorbital and parietal (0) or suture makes modest entry into supratemporal fenestra at maturity; postorbital and parietal in broad contact (1) or suture on skull table entirely (2)

150. Frontoparietal suture concavoconvex (0) or linear (1) between supratemporal fenestrae

151. Supratemporal fenestra with fossa; dermal bones of skull roof do not overhang rim at maturity (0) or dermal bones of skull roof overhang rim of supratemporal fenestra near maturity; fenestrae small, with a circular or nearly circular shape (1) or supratemporal fenestra closes during ontogeny (2) **or dermal bones of skull roof overhang rim of supratemporal fenestra near maturity; fenestrae large, significantly longer than wide, with an oval shape (3)**

The new state (3) concerns the morphology of the supratemporal fenestrae in the species of *Purussaurus*, like *P. neivensis* (see Langston, 1965, Fig. 37), *P. mirandai* (see Aguilera *et al*., 2006, Figs. 2 and 7) and also in *P. brasiliensis* (see Aguilera *et al*., 1989, Figs. 5 and 7), even though the last species is not included in this phylogenetic analysis.

In almost all Caimaninae, the dermal bones of skull roof overhang the rim of the supratemporal fenestrae, differently from what occurs plesiomorphically in Eusuchia, where the absence of the overhang allows a “fossa” to exist around the fenestrae (Brochu, 1997). The only exceptions within Caimaninae are *Culebrasuchus mesoamericanus*, in which the dermal bones do not overhang and the fossa is present (Hastings *et al*., 2013) and in the genus *Paleosuchus*, in which the fenestrae obliterate themselves during ontogeny (Brochu, 1997).

In *Purussaurus*, the overhanging of the bones in the rim of the supratemporal fenestrae is also present, but the fenestrae of this taxon is different from those of other Caimaninae due to their large size (which occupies a larger area of the skull roof than in other taxa of the group) and for being longer than wider, with a roughly oval shape. In most of the other Caimaninae, the supratemporal fenestrae has a small size with a circular or roughly circular shape, such as in *Caiman*, *Melanosuchus* and *Mourasuchus*.

152. Shallow fossa at anteromedial corner of supratemporal fenestra (0) or no such fossa; anteromedial corner of supratemporal fenestra smooth (1)

153. Medial parietal wall of supratemporal fenestra imperforate (0) or bearing foramina (1)

154. Parietal and squamosal widely separated by quadrate on posterior wall of supratemporal fenestra (0) or parietal and squamosal approach each other on posterior wall of supratemporal fenestra without actually making contact (1) or parietal and squamosal meet along posterior wall of supratemporal fenestra (2)

155. Skull table surface slopes ventrally from sagittal axis (0) or planar (1) at maturity

156. Posterolateral margin of squamosal horizontal or nearly so (0) or upturned to form a discrete horn (1) or producing a high transversely oriented eminence at the posterior margin, late in ontogeny (2). From Salas-Gismondi *et al*. (2015), character 157, modified from Brochu (2011), character 157.

157. Mature skull table with broad curvature; short posterolateral squamosal rami along paroccipital process (0) or with nearly horizontal sides; significant posterolateral squamosal rami along paroccipital process (1)

158. Squamosal does not extend (0) or extends (1) ventrolaterally to lateral extent of paraoccipital process

159. Supraoccipital exposure on dorsal skull table small (0), absent (1), large (2), or large such that parietal is excluded from posterior edge of table (3)

160. Anterior foramen for palatine ramus of cranial nerve VII ventrolateral (0) or ventral (1) to basisphenoid rostrum

161. Sulcus on anterior braincase wall lateral to basisphenoid rostrum (0) or braincase wall lateral to basisphenoid rostrum smooth; no sulcus (1)

162. Basisphenoid not exposed extensively (0) or exposed extensively (1) on braincase wall anterior to trigeminal foramen

163. Extensive exposure of prootic on external braincase wall (0) or prootic largely obscured by quadrate and laterosphenoid externally (1)

164. Laterosphenoid bridge comprised entirely of laterosphenoid (0) or with ascending process or palatine (1)

165. Capitate process of laterosphenoid orientated laterally (0) or anteroposteriorly (1) toward midline

166. Parietal with recess communicating with pneumatic system (0) or solid, without recess (1)

167. Significant ventral quadrate process on lateral braincase wall (0) or quadrate–pterygoid suture linear from basisphenoid exposure to trigeminal foramen (1)

168. Lateral carotid foramen opens lateral (0) or dorsal (1) to basisphenoid at maturity

169. External surface of basioccipital ventral to occipital condyle orientated posteroventrally (0) or posteriorly (1) at maturity

170. Posterior pterygoid processes tall and prominent (0) or small and project posteroventrally (1) or small and project posteriorly (2)

171. Basisphenoid thin (0) or anteroposteriorly wide (1) ventral to basioccipital

172. Basisphenoid not broadly exposed ventral to basioccipital at maturity; pterygoid short ventral to median eustachian opening (0) or basisphenoid exposed as broad sheet ventral to basioccipital at maturity; pterygoid tall ventral to median eustachian opening (1)

173. Exoccipital with very prominent boss on paroccipital process; process lateral to cranioquadrate opening short (0) or exoccipital with small or no boss on paroccipital process; process lateral to cranioquadrate opening long (1)

174. Lateral eustachian canals open dorsal (0) or lateral (1) to medial eustachian canal

175. Exoccipitals terminate dorsal to basioccipital tubera (0) or send robust process ventrally and participate in basioccipital tubera (1) or send slender process ventrally to basioccipital tubera (2)

176. Quadrate foramen aereum on mediodorsal angle (0) or on dorsal surface (1) of quadrate

177. Quadrate foramen aereum is small (0), comparatively large (1), or absent (2) at maturity

178. Quadrate lacks (0) or bears (1) prominent, mediolaterally thin crest on dorsal surface of ramus

179. Attachment scar for posterior mandibular adductor muscle on ventral surface of quadrate ramus forms modest crests (0) or prominent knob (1)

180. Quadrate with small, ventrally reflected medial hemicondyle (0) or with small medial hemicondyle; dorsal notch for foramen aereum (1) or with prominent dorsal projection between hemicondyles (2) or with expanded medial hemicondyle (3)

181. Orbits equal or sub equal than infratemporal fenestrae (0) or orbits larger than infratemporal fenestrae, supratemporal fenestrae smaller or obliterated (1) or orbits larger than infratemporal fenestrae (2). From Bona *et al*. (2013a), character 165. Rephrased.

182. Prefrontal-frontal not thickened or thickened forming a flange (0) or thickened forming a marked knob (1) at the anterior-medial margin of the orbits. From Bona *et al*. (2013a) character 167.

183. Dentary at level of 1st and 4th teeth equal height or teeth higher than at level of 11th-12th teeth (0) or dentary at level of 1st and 4th teeth lower than at level of 11th-12th teeth (1). From Pinheiro *et al*. (2012), character 124, rephrased from Bona (2007).

184. Anterior extremity of the frontal long and reaching or exceeding the anterior margins of the orbits (0), or short, not reaching the anterior margins of the orbits (1). From Barrios (2011) character 104, translated from Spanish.

185. Posterior margin of the skull table transversely straight to slightly concave (0) or deeply concave (1). From Barrios (2011) character 108, translated from Spanish.

186. **Preorbital crest absent (0) or present (1).** From Barrios (2011) character 109, translated from Spanish. Rephrased.

187. Jugal lateromedially slender and dorsoventrally low (0), or jugal lateromedially wide and dorsoventrally low, with a blade-like shape (1) or jugal lateromedially wide and dorsoventrally high, with a cylindrical shape (2). **New character.**

In the plesiomorphic condition in Eusuchia, present also in the external group *Bernissartia fagesii*, the jugal bone is latero-medially slender and dorsoventrally low, not exhibiting any sort of expansion in both these dimensions. In *Mourasuchus*, however, the morphology of the jugal is significantly variable. *M. atopus* and *M. arendsi* preserve the plesiomorphic condition (Fig. 8-A and D), whilst in *M. amazonensis* and UFAC-1424 the jugal is latero-medially wide, whilst keeping a dorsoventrally low profile, thus exhibiting a “blade-like” shape (Fig. 8-B and E). This morphology is also seen, convergently, in *Acynodon adriaticus*, a basal Eusuchian from the Late Cretaceous of Italy (see Delfino *et al*., 2008). And *M. pattersoni* sp. nov., in its turn, exhibits a jugal that is both latero-medially wide and dorsoventrally high, exhibiting a cylindrical shape (Fig. 8-C and F).

**S.2. List of scorings for the taxa included in the phylogenetic analysis**

The scorings of all taxa used in the analysis were based on those made available by Brochu (2011), with the exception of *Mourasuchus pattersoni* sp. nov, which were made by the authors and by Langston (2008, postcranium only) and UFAC-1424, which were made by the authors, and of the following which were available in different papers: *Mourasuchus amazonensis*, *M. atopus* and *Caiman gasparinae* (Bona, Riff & Gasparini, 2013); *Caiman brevirostris* (Fortier *et al*., 2014); *Culebrasuchus mesoamericanus* (Hastings *et al*., 2013) and *Centenariosuchus gilmorei* (Hastings *et al*., 2013; Hastings, Reisser & Scheyer, 2016); *Globidentosuchus brachyrostris* (Scheyer *et al*., 2013; Hastings, Reisser & Scheyer, 2016); *Eocaiman palaeocenicus* and *E. itaboraiensis* (Pinheiro *et al*., 2012); *Gnatusuchus pebasensis*, *Kuttanacaiman iquitosensis* and *Caiman wannlangstoni* (Salas-Gismondi *et al*., 2015).

*Bernissartia fagesii* ??????0???0111102100?00?0?000???0000?100010???0010?000???????10?0?00?001?1????000?0?0000?00030?001?????100????1?0000?000??0?00?0???000100?0?0??0?0?0010?0??00??0????????000?0000?000100?0?0

*Allodaposuchus precedens* ??????????????????????????????????????????????????????????????????????????????00010?0000001230000100?10000????0000000101030?0110000101100???0100000001100010000?????1??01?010001?000?0??0?0

*Acynodon iberoccitanus* ??????????????????????????????????????????????10104101????????0???????0?0?????00010?0000001060000100??0010????0000000101?00???200100?0100?110?0000??010100000?1?????????1???0????0100000010

*Acynodon adriaticus* ?????1?????????????01?100?1??????????010?10?????????01????????0???0??100??00??00010?000?0?10600001?0????11????00?0001101000??1?111?0?0100???0????0??010?0?0010??????????????1???01?0?0?0001

*Iharkutosuchus makadii* ??????????????????????????????????????????????10124?????????110???00??10?1????0001??00000110610001?0???011????0000001001100?012011001?100?1?0?00?0???12???100?2?????1???1000000??1101000000

*Hylaeochampsa venctiana* ????????????????????????????????????????????????????????????????????????0?????0??????????0?0?10001?00??011?0?0000000100100000?21110000120????1?0?000010100000000????1?001001000?0110?0?100?

*Eothoracosaurus mississippiensis* ??????0????????????01??000????????00?00???0????122???3??????0?????00?011?0????00120?000??1025000010??00000????0000101001000????000?000100???01???10000010?001?00????????1000100000001000000

*Eosuchus minor* ??????0???0??111???01?00?0?01???0000?000??0???1122??0300?0000?10?000001100????00120?0000?1025?000100?00000?0000000101001000??100000000110???0100?10?100100001?0100??1???1010101010031000000

*Eogavialis africanum* ????????1?????11???010??????????0?????????0???1122??03?????10?100000??1101????00120?000?01025?000100?000000000000010100100000100000000121?000100?1001001000010100?0000?0101010100000?0??000

*Gryposuchus colombianus* ????0?0???001??????01??000????????????????0???11223?030100?000100000001100????0012010000?1025?000100?0?000??0?000010100100000110000000121?000100?10011010000100000100?00121010100000?0?0000

*Gavialis gangeticus* 0200000000001111011010000000111000000?0000000011223003000000001000000011000100001301000001025?000100000000000000001010010000011000000012100001000100110100001000000000001210101000001000000

*Borealosuchus sternbergii* 0000000000110010?1001000000101000001?00???0???011020000000?00010000000100000??00020?00000001310001000100001?0?000001110100000100000111100?000100?000000100101000000?10001001100000001000000

*Borealosuchus formidabilis* 000?000?0011001001001000000101000001?000?20???0110200000?000?110000000100001??00020?0000?00231000100?0000010000000101001000001002001?11000000?00?000010100101000??????001001100000001000000

*Borealosuchus acutidentatus* ????????????????????????????0???????????????????002????????????000??????0?????00020?0?00??02310001?????000????0?0??0???????0??0020?1?1100?0?0?0??0?011010?101000???????010??1?0?000010?00?0

*Borealosuchus wilsoni* ??????0??????????1001000000101??00?1?000?20???01002??100?0?001100000101000????00020?0?0???02310001?????0001?0?0?0010100100000100200101100?000100??001101001010000?00???010011?0?000010?0000

*Planocrania hengdongensis* ????????????????????1?????????????????????????1110???1????????1????0?01?0?????20010?0?????01300??1????0?00????0????01001???????0?0?1?1100????????0?0?00100?0100?????1???1???10?10001??01000

*Planocrania datagensis* ??????????????????????????????????????????????11?????0????????????????????????20010?000??10030000100??0?00???????000??01???????0?0?1?1110????????0?000010010??0?????????????10??0?0??00?00?

*Boverisuchus magnifrons* ?????????????0?0???01?000?0??1??0100?1???1????11102000??????0?2????0??1?1?????21?20?00000000300011???00000????0000001001010??100000111110?0?0?00?0?010010?10100?????????1???10000002?00?0?0

*Boverisuchus vorax* ????0?0???01001001?01?00000111??0100?10???1???1110?000?????0??1000001?1?0?????21010?0000?00030001100?00000??0?000000100100000100000111110???0100?010000100101000???01??1100110000002?000000

*Asiatosuchus germanicus* 001?0?0?1?001010?0101?000?1111??1??0??????1???11102000?????00110000??0101?00??00010?0000?0001000010???1000??0?00010???010000010000?111100???0100?001110100101000???????1100?10000003100?000

*Crocodylus affinis* 001001001010001000011100001111001100?10???1???111021010100000110000000101100??00110?0000100110000100?01000??0?000100110100000100000111100?0?010??0012001001010001??0?1?1100010000003?0?0000

*Crocodylus acer* ??????????????????????????????????????????????????????????????????????????????00110?0000?10210000100??00001???000100?10100000100010111100?002?01?00?2001001010001??0??1110001000000310?0000

*Kentisuchus spenceri* ??????0????????????????????????????????????????1?????0?????0??1000?1101?11????00110?0000?10210000100???000??0?010010100100?0011000?111110??????0?0??20010?10100???????111?0?100?0?03?0?000?

*Thecachampsa americana* ??????????????1????01?000?1111??1100?00???1???1122???4?????00010?0031?1010????00120?0000110210000100?10000????0000100001000???1000?011110?000?10?10120010010101???0????11100100?2003?00010?

*Tomistoma schlegelii* 02100000100010100010110001111110110011013010101122?1040000010010000000101000100012000000110210000101010000110100010010010000011000011111000001101001210100101000110011111100100000031000000

*Kambara implexidens* ??????0????????????01?????11????1100?10???1???11102101?????001100001101011????00110?00001102100001000100001?010000001001000?0100000111110?002011?00120010010100011101?111100100000010000000

*Australosuchus clarkae* ??????0???????1??0??1?????11???????0?10???1???1110?101?????001100001101011????00110?00001102100001000100001?0?000000????????01000?0111110?002011?01120010010100011??11?11?001000?0011?00000

*Trilophosuchus rackhami* ??????????????????????????????????????????????????????????????????????????????0?????0??????2?0??0??0?1000???0?1?000001010???01?0??0111110??12011101121010000102011101?111?0?1000?0011??00?0

*Crocodylus megarhinus* ??????0???????????001?????????????????????????11102101?????00110000??01011????00110?0000?0023000010001?000??0?000000110100000120000111110?002?11?0012?01001010?01100???11100100000031??00??

*Rimasuchus lloydi* ?????????????????????????????????????????????????????1??????????????????1?????00110?0000?00210001100?10000110100000????10?????1000?111110?001011?001200100101100111?1??1110?1000?003?0??000

*Voay robustus* ??????0????????0???011?????111??1110??????1???1110210101000001100011111011????00110?0000?002100011000100001101000101110101000100000111110?0010111001201100111110?1101?111100100000031000000

*Osteolaemus tetraspis* ??1?000010001010100011100111112011101111111010110021010100010110000110101110101010000010100210001100010000110110010111010100010001011111010010101001211100101100111011111101100000031000000

*Osteolaemus osborni* ??1?00001?00101010001110011111201110?111111???110021010100000110000110101110??101100001010021000110001000011011001001001010?01000001111101001010100121110010110011101?111101100000031000000

*Mecistops cataphractus* 10?0010010000010000011100111112012001111101010111041010100010010001110101?10100012000000100210000100010000110100001011010000010000011111000010111001200100101000111011111100100000031000000

*Crocodylus porosus* 1110000010001010101011100011112012001111201010110021010100010110001110101110100011000000100210001100110000110100100011010000010000011111000010111001200100101000111011111100110000031000000

*Crocodylus acutus* 0010000010101011001011100111112012001110201010110021010100010110001110101110100011000000100210100100110000110100100011010000011000011111000010111001200100101000111011111100110000031000000

*Crocodylus niloticus* 1010000010101010001011100111112012001111201010110021010100010110001110101110100011000000100210000100110000110100100011010000011000011111000010111001200100101000111011111100110000031000000

*Prodiplocynodon langi* ??????????????????????????????????????????????????????????????????????????????00110?0000??0030000100??10001??000010011?1000001???0?111100???0?0??0?11?010?1010001??01?0110011000000310??000

*Leidyosuchus canadensis* ????0?0???????1????010000011?1??10?0?11??11???0110?00000?0?01?100000011101????00010?00000000300001000001001000100001110100000100010111100?010100100100010010100000001?001001100100011000000

*Diplocynodon deponiae* 100?0?0???????0????01?0???1?????14?0?10??21????1?02??0?????01?10??0??1110?????001???000??010300001?0????00?????0?0001?110000010001?111100?110100?0??011?0?101?00????????1???1??100011?000?0

*Diplocynodon darwini* 100001001?010000?00010000?1111??1400?101121???011020010??0?01?10000011110100??00020?0000?01030000100?0??00??0?0000001111?000010001?111100?110100?011010101101000???????01001100100010000000

*Diplocynodon ratelii* ??????0??????000???010?00?1111001400?10??21???01002101?????0111000001?1101????00120?0000001230000100010100??0?000000111100000100010111100??1010010000001011010000000101010011001000100?00?0

*Diplocynodon hantoniensis* 100???1?1?01000010001000011111??1400?101?21???011021010????011100000111101????00120?0?000?11300?0100?101001?0?000000101100000100010111100?110100?001010101101000??0?10?010011?010001000?0?0

*Diplocynodon muelleri* ????????????????????1?01??1?????14?0?10??21???01002??2?????01110??10011100????00120?1000001230000100010100????0000011111000?010001?1?1100?110??0?01?01010110100????????01001100100011000000

*Diplocynodon tormis* ????????????????????1?????1??????????10??21???01?021?1?????01?1?00????11??????0?120??000001230000100?101001???000000111100???100010111100?110100000101010110100?0???1?001?0110010001?0?0?0?

*Ceratosuchus burdoshi* ???????????????????????????????????0?1????????11111??0?????01?10??00?11?01????00010?0?01?01020000100???100??0????00???01?????1??0??111100????????0????010?111???????????10??10010001?00?00?

*Navajosuchus mooki* ??????0?1??????0???0?00???1111??1??0?111111???11111010?????01?10??00??110?????00010?0001?010200001???0?100??0???00?00?11100?0110011111100???020??0112?0102101000????1??010??10010001100??00

*Hassiacosuchus haupti* 001?1?0?11?????0???01?000?1111?????0??11?11???111110?0?????01?10??0??11101????00010?00???010?00001??????001?0??????0?????????11001?111100?1?0200?01120010210101????????01???100100011??0??0

*Allognatosuchus polyodon* ??????????????????????????????????????????????11111010?????01?11??00?111?1????00010?000??010200001?0?????0??0?00?00?0?11100??1?011?111100????????0??20010?101???????????1???1??1???1??00?00

*Allognatosuchus wartheni* ????1?0????????????0?0000?1111??1000?11???1???11111010?100?011110000011101????00010?0000?010200001000001001?0?000000?0111001?110111111100?110200?0112001021010000?1?1?0010011001?001???00??

*Procaimanoidea kayi* ????110?1??????0???010?00?1111??10?0?112121?????010?1??100?01?11000001110?????10?????0???010?00001???0?100??0?0000000011100101101111111000??0200?011200102101010???????01001100100011000000

*Procaimanoidea utahensis* ??????????????????????????????????????????????110100?00??01011110??0011101????10110?0100?01020?00100??0100????0000000?11100?011011?111100?1?0200?01120010?101000???????01001100100011000000

*Arambourgia gaudryi* ??????????????????????????????????????????????11010??0?????01?100?0??1110?????1001??010??010200001?0???1001000??000????11?01?11011?111100?1102?0?0?1210?0210100?????????10011?010?011??00?0

*Wannaganosuchus brachymanus* ????1?0???1?00?0???010000?1111001000?11???1???111110?0?????0??100?00?11?0?????00110?0000??10200??100???100??0?100000???1??0??11011?111100????????0?12001??101000????????10011?010001?0000?0

*Alligator sinensis* 1010111011110010100010110111110011001112111112110000120?001011200000111101??010010000100001020000100000100110010000001111002011011111111001102001011200102101010001110001001100100011000000

*Alligator mississippiensis* 1010110011010010000010110111110011001112101110110001120100101120001011110100010010000100001020000100000100111000000001111002011011111111001102001011210102101010001110001001100100011000000

*Alligator thomsoni* ???????????????????01????????1?????0?1????????110000?2?????011200010011101????00100?010000102000?10??001001?1?000000????1?????10111111110?1?0200101?21010210101?001?1??01001100100011?000?0

*Alligator prenasalis* 10001?0?11????10?0?01000011111??1000?112111???11111010?????01110000011110100??00000?0100?01020000100?001001100100000011110020110111111100?110200?011200102101000001?1?001001100100011000000

*Alligator mefferdi* ???????????????????????????1?????????11???????110000120100001120001011110100??00100?0100?01020000100?0?1001?1?0000000?1110020110111111110?110200?011210102101010??1???001001100100011000000

*Alligator olseni* ??????0?1?????10???01?10011111???100?11???????11010010?????011200000011101????00100?01000?10200??100???100??0?0?0001011111020110111111110?110200?011200102101010?????000100?1?01000110000?0

*Alligator mcgrewi* 1000100011010010?00010000111?1101??0?11???1???11110010?100?011100000111101????00000?0100?01020000100?001001?001000000111100201101111111000110200?011200102101010?01???001001100100011000000

*Stangerochampsa mccabei* ????110???010010?0001000001111001000?01??11???111010100????0111110000?110?????00110?0002?11020000100?00100????000000011110010120011111100?110200?0?1100102101000??1?1??01001100100011?000?0

*Albertochampsa langstoni* ??????????????????????????????????????????????????????????????????????????????001?0?0?0??110200001?0??01?0????00??000111100?0?2001?111100???0?0??0?110010210100????????010011001?00010?0000

*Brachychampsa montana* 10101100101100???0001??000111100?000?103111???11101101?????01110?00001110100??00110?0002?11010000100?001001?0?010000011110010120011111100?110200?01110010110102000101?001001100100010000000

*Brachychampsa sealeyi* ?????????????????????????????????????10???1???11101??0???????11??0000111?1????001?0?0002?1101000010???010????????00?????????0?200?1101?00???????????100???????2?????????????1??10??1?0?0???

*Purussaurus mirandai* ????????????????????1?11001?????11?0?1????????1100?0?2??1?1??1201100?11?01????01112?0000001020010100??0100????00000001111011113110?111110?11020?1011203???11102?????????10011?2?00?10001110

*Purussaurus neivensis* 101?100?11000010?0??1????011???????0?11???1???1?00??1?1010?011201100011001????0?112?0000001020000100?001001?0?0?0000011110111131101111110?110201?0112031?211102000101??010??102100010001110

*Caiman yacare* 1011110011100010000010101111110011001111221111110021121010101010110201100101010011000000201120000100000100110010000001111011111101111111001102011011201112101030001010001001102100011000010

*Caiman crocodilus* 1011110011100010000010101111110011001111221111110021121010101010110201100101010011000000201120000100000100110010000001111011111001111111001102011011201112101030001010001001102100011000010

*Caiman latirostris* 101110001110001000001010?111110011001111221121110021121010101110110201100???010011000000201020010100000100110010000001111011111001111111001102011011211112101030001010001001102100011000010

*Melanosuchus niger* 10111100111?00100000101011111100110011112211211100211210101011101102011001??010011000000201020010110000100110010000001111011111001111111001102011011211112101030001010001001102100011000010

*Paleosuchus trigonatus* 1001111111010010100010001111112113001111321112110021222111101110110001100101011011000010?01020000100000100110001000101111011110001111111011102011011212112101020001010001001102100011000000

*Paleosuchus palpebrosus* 1001111111010010101010001111112113001111321112110021222111101110110001100?01011011000010?01020000100000100110001000101111011110001111111011102011011212112101020001010001001102100011000000

*Caiman brevirostris* ??????????????????????????????????????????????1100?112????????1???0201??????????110??0???11020?10100???????????0?0000??1?0????1001?????10???????1?1?201????01??????????0??????210??11000?10

*Tsoabichi greenriverensis* ???????????????????01????????????????10??20???1100???2????????1??????11???01??00010?10????10??0001????????????????????????????0001?111110?1?0?0??0??211???101?2????????????????100??1000000

*Centenariosuchus gilmorei* ??????????????????????????????????????????????11002??2??????????????????????????1?0??0002??02??0??0???????????0????????????????????????1????????10112?111?10103?????????1???1?210?01????0??

*Culebrasuchus mesoamericanus* ??????????????????????????????????????????????1??2???2?????0??10????????????????1?????????1000?0????????????0??????????????????????????0???????????1??010210??3????????01?0?1?0??????0?00?0

*Globidentosuchus brachyrostris* ???????????????????01?????????????????????????11101??0?11??0?1101103111011????0012??000??01?000001???1?10???????0???0?11?????1?1???1?1100????????0??2011??101?3?????????1???1?????0?1?010?0

*Eocaiman cavernensis* ??????????????????????????????????????????????1010???2??????111???????????????00???????????????????????100??0?1?00000???????01?????1?1??0?????0???????????????????????????????????????1????

*Eocaiman palaeocenicus* ??????????????????????????????????????????????1110???2?????0??10?100?1100?????????????????????????????????????????????????????????????????????????????????????????????????????????????1????

*Eocaiman itaboraiensis* ??????????????????????????????????????????????1010?????????????????????????????0??????????????????????????????????????????????????????????????????????????????????????????????????????1????

*Gnatusuchus pebasensis* ??????????????????????????????????????????????2020?12000?01011101000011001????02110?01001010000001???0010?????1?????11?1001101?0?1???1100???0?0?1?0120111?10103?????1??0100110210011?0?1000

*Kuttanacaiman iquitosensis* ??????????????????????????????????????????????1110211200?0101110?102011001????10110?00001010200001?0?00100????1000010111101?11?1001??1100???020?10?121111210103?????1??0100?1021000110?10?0

*Caiman wannlangstoni* ??????????????????????????????????????????????11102112????????10??????????????00010?00???0102001010??0?1001?0?1?0???011111??11110??111110?110?0110112011??10103?????1??010??1021000110?1010

UFAC-1424 ???????????????????????????????????????????????????????????0????????0????1??????????????????????????????????????0???0111?????1?1?1?????1??1?0?01???12111??12103?????????????1??100?1???10?1

*Mourasuchus amazonensis* ??????????????????????????????????????????????1132????????????????????????????10121?00?0?1???0???1???????????????0????????????311?111111??11????????2111????1?3?????????????1???????210?0?1

*Mourasuchus arendsi* ?????1??11????1??0?????011????????????????????11?2???????????????????1100????????20?02000110?0000100???100??????00?0??1??????1?1??1??1?10?1?0?01????2?1???1?103?????????1???1??100?1210?000

*Mourasuchus atopus* ???????????????????????0??11????1300??????????11323112?????01??0?100011000????101????200?????0?001????0?0?????0??00?01111011?1?1???1?1110???????????21?1??????3?????????10??????????2101??0

***Mourasuchus pattersoni* ??????????????10?00??110?1???????????????????????2?????????0??20?1????1?????????1?1??0000??0?000?1?0???10?????0????00?11????11????????????1?0?0?????????????10??????1???1??1?02??00???0???2**

**S.3. List of sources consulted for scoring of characters by taxon**

This list shows the sources used by the authors to score the morphological characters of the phylogenetic analysis for each taxon. Taxa were scored based on direct observations of fossil or extant osteological specimens, images from published literature, other photos taken of the specimens, or a combination of these. For the taxa that were directly observed or scored through unpublished photographs, the catalogue number of the specimens consulted is provided; for scorings based on photographs available in published papers, the citation of the paper where the images were found is provided.

*Acynodon adriaticus*: Delfino, Martin & Buffetaut (2008).

*Acynodon iberoccitanus*: Buscalioni, Ortega & Vasse (1997); Martin (2007).

*Albertochampsa langstoni*: SMM P. 67.15.3.

*Alligator mississipiensis*: AMNH 71621.

*Alligator sinensis*: AMNH 23898; AMNH 23907.

*Alligator mcgrewi*: Schmidt (1941).

*Alligator olseni*: White (1942).

*Alligator mefferdi*: Mook (1946).

*Alligator prenasalis*: Mook (1932).

*Alligator thomsoni*: Mook (1923).

*Allodapasuchus precedens*: Buscalioni *et al*. (2001); Delfino *et al*. (2008b); Martin (2010).

*Allognathosuchus polyodon*: Brochu (2004a); Mook (1921a).

*Allognathosuchus wartheni*: Case (1925); Bartels (1983).

*Arambourgia gaudryi*: Brochu (1999).

*Asiatosuchus germanicus*: Berg (1966).

*Australosuchus clarkae*: Willis & Molnar (1991).

*Bernissartia fagesii*: Buffetaut (1975); Norell & Clark (1990).

*Borealosuchus acutidentatus*: Brochu (1997a).

*Borealosuchus formidabilis*: Erickson (1976).

*Borealosuchus sternbergii*: Gilmore (1910); Brochu (1997a).

*Borealosuchus wilsoni*: AMNH 7637; Mook (1959).

*Boverisuchus magnifrons*: Brochu (2013).

*Boverisuchus vorax***:** Langston (1975); Brochu (2013).

*Brachychampsa montana*: AMNH 5032; Norell, Clark & Hutchinson (1994).

*Brachychampsa sealeyi*: Williamson (1996).

*Caiman brevirostres*: UFAC 196; UFAC 5388; Fortier *et al*. (2014).

*Caiman crocodilus*: AMNH R 43291; AMNH R 73048; AMNH R 137179.

*Caiman gasparinae*: Bona & Carabajal (2013).

*Caiman latirostris*: AMNH R 28367; AMNH R 143183.

*Caiman wannlangstoni*: Salas-Gismondi *et al*. (2015).

*Caiman yacare*: AMNH R 97305.

*Centenariosuchus gilmorei*: Hastings *et al*. (2013); Hastings, Reisser & Scheyer (2016).

*Ceratosuchus burdoshi*: Bartels (1984).

*Crocodylus acer*: Mook (1921b).

*Crocodylus acutus*: AMNH R 9659.

*Crocodylus affinis*: Mook (1921c).

*Crocodylus megarhinus*: Mook (1927).

*Crocodylus niloticus*: AMNH 10081.

*Crocodylus porosus*: AMNH R 66378.

*Culebraushcus mesoamericanus*: Hastings *et al*. (2013).

*Diplocynodon darwini*: HLMD-Me 7500; Brochu (1999).

*Diplocynodon deponiae*: HLMD-Be 147; Delfino & Smith (2012).

*Diplocynodon hantoniensis*: AMNH 27632; Owen (1850); Brochu (1999); Piras & Buscalioni (2006).

*Diplocynodon muelleri*: Piras & Buscalioni (2006).

*Diplocynodon ratelii*: MNHN.F SG 539; Brochu (1997b).

*Diplocynodon tormis*: Buscalioni, Sanz & Casanovas (1992).

*Eocaiman cavernensis*: AMNH 3158; Simpson (1933).

*Eocaiman itaboraiensis*: MCT 1791-R; MCT 1792-R; MCT 1793-R; MCT 1794-R; Pinheiro *et al*. (2012).

*Eocaiman palaeocenicus*: MPEF - PV 1933; Bona (2007).

*Eogavialis africanum*: SMNS 11785.

*Eosuchus minor*: Brochu (2006).

*Eothoracosausus mississipiensis*: Brochu (2004b).

*Gavialis gangeticus*: AMNH R 88316.

*Globidentosuchus brachyrostris*: Scheyer *et al*. (2013); Hastings, Reisser & Scheyer, 2016.

*Gnatusuchus pebasensis*: Salas-Gismondi *et al*. (2015).

*Gryposuchus colombianus*: UCMP 41136; UCMP 38358; UCMP 39389; Langston (1965).

*Hassiacosuchus haupti*: Brochu (2004a).

*Hylaeochampsa vectiana*: Clark & Norell (1992); Brochu (1997b).

*Iharkutosuchus makadii*: Ösi, Clark & Weishampel (2007).

*Kambara implexidens*: Sallisbury & Willis (1996).

*Kentisuchus spenceri*: Brochu (2007).

*Kuttanacaiman iquitosensis*: Salas-Gismondi *et al*. (2015).

*Leidyosuchus canadensis*: AMNH 5352; Sternberg (1932).

*Mecistops cataphractus*: AMNH R 29300.

*Melanosuchus fisheri*: MCNC 243; Medina (1976).

*Melanosuchus niger*: AMNH R 58130.

*Mourasuchus amazonensis*: DGM 526-R; Price (1964).

*Mourasuchus arendsi*: CIAAP-1297; Bocquentin-Villanuvea (1984).

*Mourasuchus atopus*: UCMP 38012; Langston (1965).

*Mourasuchus pattersoni* sp. nov.: MCNC-PAL-110-72V.

*Navajosuchus mooki*: AMNH 6780; Simpson (1930).

*Necrosuchus ionensis*: AMNH 3219; Brochu (2011).

*Orthogenysuchus olseni*: AMNH 5178; Mook (1924).

*Osteolaemus osborni*: AMNH R 160900.

*Osteolaemus tetrapsis*: YPM HER R 16944.

*Paleosuchus palpebrosus*: AMNH R 93812; AMNH R 97326.

*Paleosuchus trigonatus*: AMNH R 58136; AMNH R 66391.

*Planocrania datagensis*: Brochu (2013).

*Planocrania hengdongensis*: Brochu (2013).

*Procaimanoidea kayi*: Mook (1941a).

*Procaimanoidea utahensis*: Gilmore (1946).

*Prodiplocynodon langi*: Mook (1941b).

*Purussaurus mirandai*: UNEFM-CIAAP-1369; Aguilera, Riff & Bocquentin-Villanueva (2006).

*Purussaurus neivensis*: UCMP 39704; Langston (1965).

*Rimasuchus lloidy*: Fourtau (1920).

*Stangerochampsa maccabei*: Wu, Brinkman & Russell (1996).

*Thecachampsa americana*: Sellards (1915); Mook (1921d).

*Thoracosaurus neocesariensis*: Carpenter (1983).

*Tomistoma schlegelli*: AMNH 15177.

*Trilophosuchus rackhami*: Willis (1993).

*Tsoabichi greenriverensis*: Brochu (2010).

UFAC-1424: Bocquentin & Souza-Filho (1990); Bona, Degrange & Fernández (2013); personal analysis of the specimen.

*Voay robustus*: AMNH 3101; AMNH 3104; Brochu (2007b).

*Wannaganosuchus brachymanus*: Erickson (1982).

**S.4. The detailed phylogeny of Eusuchia obtained in this work**

Figure S1.

**S.5. Supplementary Bibliography**

Aguilera OA, Riff D, Bocquentin-Villanueva J. 2006. A new giant *Purussaurus* (Crocodyliformes, Alligatoridae) from the upper Miocene Urumaco Formation, Venezuela. *Journal of Systematic Palaeontology* 4:221–232.

Bartels WS. 1983. A transitional Paleocene-Eocene reptile fauna from the Bighorn Basin, Wyoming. *Herpetologica* 39(4):359–374.

Bartels WS. 1984. Osteology and systematic affinities of the horned alligator *Ceratosuchus* (Reptilia, Crocodilia). *Journal of Paleontology* 58:1347–1353.

Berg DE. 1966. Die Krokodile, insbesondere *Asiatosuchus* und aff. *Sebecus*?, aus dem Eozän von Messel bei Darmstadt/Hessen. *Abhandlungen des Hessischen Landesamtes für Bodenforschung* 52:1–105.

Bocquentin-Villanueva J. 1984. Um nuevo Nettosuchidae (Crocodylia, Eusuchia)

proveniente de la Formación Urumaco (Mioceno Superior), Venezuela. *Ameghiniana* 21:3–8.

Bocquentin J, Souza-Filho JP. 1990. O crocodiliano sul-americano *Carandaisuchus* como sinonímia de *Mourasuchus* (Nettosuchidae). *Revista Brasileira de Geociências* 20:230–233.

Bona P. 2007. Una nueva especie de *Eocaiman* Simpson (Crocodylia, Alligatoridae) del Paleoceno Inferior de Patagonia. *Ameghiniana* 44(2):435–445.

Bona P, Carabajal, AP. 2013. *Caiman gasparinae* sp. nov., a huge alligatorid (Caimaninae) from the late Miocene of Paraná, Argentina. *Alcheringa* 37(4):462–473.

Bona P, Degrange FJ, Fernández MS. 2013. Skull anatomy of the bizarre Crocodylian *Mourasuchus nativus* (Alligatoridae, Caimaninae)**.** *The Anatomical Record* **296**(2):227–239.

Brochu CA. 1997a. A review of ‘*Leidyosuchus*’ (Crocodyliformes, Eusuchia) from the Cretaceous through Eocene of North America. *Journal of Vertebrate Paleontology* 17:679–697.

Brochu CA. 1997b. Phylogenetic Systematics and Taxonomy of Crocodylia. PHD Thesis, University of Texas.

Brochu CA. 1999. Phylogenetics, taxonomy, and historical biogeography of Alligatoroidea. *Memoir of the Society of Vertebrate Paleontology* 6:9–100.

Brochu CA. 2004a. Alligatorine phylogeny and the status of *Allognathosuchus* Mook, 1921. *Journal of Vertebrate Paleontology* 24:856–872.

Brochu CA. 2004b. A new gavialoid crocodylian from the Late Cretaceous of eastern North America and the phylogenetic relationships of thoracosaurs. *Journal of Vertebrate Paleontology* 24:610–633.

Brochu CA. 2006. Osteology and phylogenetic significance of *Eosuchus minor* (Marsh 1870), new combination, a longirostrine crocodylian from the Late Paleocene of North America. *Journal of Paleontology* 80:162–186.

Brochu CA. 2007a. Systematics and taxonomy of Eocene tomistomine crocodylians from Britain and Northern Europe. *Palaeontology* 50(4):917–928.

Brochu CA. 2007b. Morphology, relationships and biogeographic significance of an extinct horned crocodile (Crocodylia, Crocodylidae) from the Quaternary of Madagascar. *Zoological Journal of the Linnean Society* 150:835–63.

Brochu CA. 2010. A new alligatoroid from the Lower Eocene Green River Formation of Wyoming and the origin of caimans. *Journal of Vertebrate Paleontology* 30:1109–1126.

Brochu CA. 2011. Phylogenetic relationships of *Necrosuchus ionensis* Simpson, 1937 and the early history of caimanines. *Zoological Journal of the Linnean Society* 163:S228–S256.

Brochu CA. 2013. Phylogenetic relationships of Palaeogene ziphodont eusuchians and the status of *Pristichampsus* Gervais, 1853. *Earth and Environmental Science Transactions of the Royal Society of Edinburgh* 103:521–550.

Buffetaut E. 1975. Sur l'anatornie et la position systematique de *Bernissartia fagesii* Dollo. *L..* 1883, crocodilien du Wealdien de Bernissart, Belgique. *Bulletin de 1'Institut Royal des Sciences Naturelles de Belgique (sci. Terre)* 51:1–20.

Buscalioni AD, Sanz JL, Casanovas ML. 1992. A new species of the eusuchian crocodile *Diplocynodon* from the Eocene of Spain. *Neues Jahrbuch für Geologie und Palaontologie Abandlungen* 187(1):1–29.

Buscalioni AD, Ortega F, Vasse D. 1997. New crocodiles (Eusuchia: Alligatoroidea) from the Upper Cretaceous of southern Europe*.* *Comptes Rendus de l’Academie des Sciences de Paris, Sciences de la Terre et des Planétes* 325:525–530.

Buscalioni AD, Ortega F, Weishampel DB, Jianu CM. 2001. A revision of the crocodyliform *Allodaposuchus precedens* from the Upper Cretaceous of the Hateg Basin, Romania. Its relevance in the phylogeny of Eusuchia. *Journal of Vertebrate Paleontology* 21:74–86.

Carpenter K. 1983. *Thoracosaurus neocesariensis* (De Kay, 1842) (Crocodylia: Crocodylidae) from the Late Cretaceous Ripley Formation of Mississippi. *Mississippi Geology* 4(1):1–10.

Case EC. 1925. Note on a new species of the Eocene crocodilian *Allognathosuchus, A. wartheni. Contributions from the Museum of Geology, University of Michigan* 2:93–97.

Clark JM, Norell MA. 1992. The Early Cretaceous crocodylomorph *Hylaeochampsa vectiana* from the Wealden of the Isle of Wight. *American Museum Novitates* 3032:1–19.

Delfino M, Smith T. 2012. Reappraisal of the morphology and phylogenetic relationships of the middle Eocene alligatoroid *Diplocynodon deponiae* (Frey, Laemmert, and Riess, 1987) based on a three-dimensional specimen. *Journal of Vertebrate Paleontology* 32(6):1358–1369. DOI:10.1080/02724634.2012.699484.

Delfino M, Codrea V, Folie A, Dica P, Godefroit P, Smith T. 2008. A complete skull of *Allodaposuchus precedens* Nopcsa 1928 (Eusuchia) and a reassessment of the morphology of the taxon based on the Romanian remains. *Journal of Vertebrate Paleontology* 28:111–122.

Delfino M, Martin JE, Buffetaut E. 2008. A new species of *Acynodon* (Crocodylia) from the Upper Cretaceous (Santonian-Campanian) of Villaggio del Pescatore, Italy. *Palaeontology* 51:1091–1106.

Erickson B.R. 1976. Osteology of the early eusuchian crocodile *Leidyosuchus formidabilis,* sp. nov. *Monographs of the Science Museum of Minnesota (Paleontology)*, 2, 1–61.

Erickson BR. 1982. *Wannaganosuchus*, a new alligator from the Paleocene of North America. *Journal of Paleontology* 56(2):492–506.

Fortier DC, Souza-Filho JP, Guilherme E, Maciente A, Schultz CL. 2014. A new specimen of *Caiman brevirostris* (Crocodylia, Alligatoridae) from the Late Miocene of Brazil. *Journal of Vertebrate Paleontology* 34(4):820–834.

Fourtau R. 1920. *Contribution à l'etude des vertebres miocènes de l'Egypte*. Cairo: Egypt Survey Department.

Gasparini, Z. 1985. Un Nuevo cocodrilo (Eusuchia) Cenozoico de América del Sur. *Coletânea de Trabalhos Paleontológicos MME–DNPM, série Geologia* 27:51–53.

Gilmore CW. 1910. *Leidyosuchus sternbergii*, a new species of crocodile from the Cretaceous Beds of Wyoming. *Proceedings of the United States National Museum* 38(1762):485–502.

Gilmore CW. 1946. A new crocodilian from the Eocene of Utah. *Journal of Paleontology* 20:62–67.

Hastings AK, Bloch JI, Jaramillo CA, Rincon AF, MacFadden BJ. 2013. Systematics and biogeography of crocodylians from the Miocene of Panama. *Journal of Vertebrate Paleontology* 33(2):239–263.

Hastings AK, Reisser M, Scheyer TM. 2016. Character evolution and the origin of Caimaninae (Crocodylia) in the New World Tropics: new evidence from the Miocene of Panama and Venezuela. *Journal of Paleontology* 90(2):317–332. DOI:10.1017/jpa.2016.37

Langston W. 1965. Fossil crocodilians from Colombia and the Cenozoic history of the Crocodilia in South America. *University of California Publications in Geological Sciences* 52:1–168.

Langston W. 1975. Ziphodont crocodiles: *Pristichampsus vorax* (Troxell), New Combination, from the Eocene of North America. *Fieldiana: Geology* 33(16):291–314.

Martin JE. 2007. New material of the Late Cretaceous globidontan *Acynodon iberoccitanus* (Crocodylia) from southern France. *Journal of Vertebrate Paleontology* 27:362–372.

Martin JE. 2010. *Allodaposuchus* Nopsca, 1928 (Crocodylia, Eusuchia), from the Late Cretaceous of southern France and its relationships to Alligatoroidea. *Journal of Vertebrate Paleontology* 30:756–767.

Medina CJ. 1976. Crocodilians from the Late Tertiary of Northwestern Venezuela: *Melanosuchus fisheri* sp. nov. *Breviora* 438:1–14.

Mook CC. 1921a. *Allognatosuchus,* a new genus of Eocene crocodilians. *Bulletin of the American Museum of Natural History* 44:105–110.

Mook CC. 1921b. The skull of *Crocodilus acer* Cope. *Bulletin of the American Museum of Natural History* 44:117–121.

Mook CC. 1921c. Description of a skull of a Bridger crocodilian. *Bulletin of the American Museum of Natural History* 44:111–116.

Mook CC. 1921d. Skull characters and affinities of the extinct Florida gavial *Gavialosuchus americana* (Sellards). *Bulletin of the American Museum of Natural History* 44:33–41.

Mook CC**.** 1923. A new species of *Alligator* from the Snake Creek Beds. *American Museum Novitates* 73:1–13.

Mook CC. 1924. A new crocodilian from the Wasatch Beds. *American Museum Novitates* 137:1–4.

Mook CC. 1927. The skull characters of *Crocodylus megarhinus* Andrews. *American Museum Novitates* 289:1–8.

Mook CC. 1932. A study of the osteology of *Alligator prenasalis* (Loomis). *Bulletin of the Museum of Comparative Zoology* 74:19–41.

Mook CC. 1941a. A new fossil from Colombia. *Proceedings of the United States*

*National Museum* **91**(31–22):55–61.

Mook CC. 1941b. A new crocodilian, *Hassiacosuchus kayi*, from the Bridger Eocene Beds of Wyoming. *Annals of the Carnegie Museum* 28:207–220.

Mook CC. 1946. A new Pliocene alligator from Nebraska. *American Museum Novitates* 1311:295–304.

Mook CC. 1959. A new species of fossil crocodile of the genus *Leidyosuchus* from the Green River Beds. *American Museum Novitates* 1933:1–6.

Norell MA, Clark JM. 1990. A reanalysis of *Bernissartia fagesii*, with comments on its phylogenetic position and its bearing on the origin and diagnosis of the Eusuchia. *Bulletin de l’Institut Royal des Sciences Naturelles de Belgique* 60:115–128.

Norell MA, Clark JM, Hutchison JH. 1994. The Late Cretaceous alligatoroid *Brachychampsa montana* (Crocodylia): new material and putative relationships. *American Museum Novitates* 3116:1–26.

Ösi A, Clark JM, Weishampel DB. 2007. First report on a new basal eusuchian crocodyliform with multicusped teeth from the Upper Cretaceous (Santonian) of Hungary. *Neues Jahrbuch für Geologie und Paläontologie Abhandlungen* 243:169–177.

Owen R. 1850. *Monograph on the Fossil Reptilia of the London Clay, and of the Bracklesham and Other Tertiary Beds, Part II: Crocodilia (Crocodilus, etc.)*. London: Paleontographical Society.

Pinheiro AEP, Fortier DC, Pol D, Campos DA, Bergqvist, LP. 2012. A new *Eocaiman* (Alligatoridae, Crocodylia) from the Itaboraí Basin, Paleogene of Rio de Janeiro, Brazil. *Historical Biology: an International Journal of Paleobiology* 25:327–337. DOI: 10.1080/08912963.2012.705838.

Piras P, Buscalioni AD. 2006. *Diplocynodon muelleri* comb. nov., an Oligocene diplocynodontine alligatoroid from Catalonia (Ebro Basin, Lleida Province, Spain). *Journal of Vertebrate Paleontology* 26:608–620.

Price LI. 1964. Sobre o crânio de um grande crocodilídeo extinto do Alto de Rio Juruá, Estado do Acre. *Anais da Academia Brasileira de Ciências* 56:59–66.

Salas-Gismondi R, Flynn JJ, Baby P, Tejada-Lara JV, Wesselingh FP, Antoine P-O. 2015 A Miocene hyperdiverse crocodylian community reveals peculiar trophic dynamics in proto-Amazonian mega-wetlands. *Proceedings of the Royal Society B* 282:20142490. DOI: http://dx.doi.org/10.1098/rspb.2014.2490

Salisbury SW, Willis PMA. 1996. A new crocodylian from the Early Eocene of southeastern Queensland and a preliminary investigation of the phylogenetic relationships of crocodyloids. *Alcheringa* 20:179–227.

Scheyer TM, Aguilera OA, Delfino M, Fortier DC, Carlini AA, Sánchez R, Carrillo-Briceño JD, Quiroz L, Sánchez-Villagra M.R. 2013. Crocodylian diversity peak and extinction in the late Cenozoic of the northern Neotropics. *Nature Communications* 4:1907. DOI: http://dx.doi.org/10.1038/ncomms2940.

Schimidt KP. 1941. A new fossil alligator from Nebraska. *Fieldiana: Geology* 8:27–32.

Sellards EH. 1915. A new gavial from the Late Tertiary of Florida. *American Journal of Science* 40:135–138.

Simpson GG. 1930. *Allognathosuchus mooki*, a new crocodile from the Puerco Formation. *American Museum Novitates* 445:1–16.

Simpson GG. 1933. A new crocodilian from the *Notostylops* Beds of Patagonia. *American Museum Novitates* 623:1–9.

Sternberg CM. 1932. The skull of *Leidyosuchus canadensis* Lambe. *American Midland Naturalist* 13:57–168.

White TE. 1942. A new alligator from the Miocene of Florida. *Copeia* 1942:3–7.

Williamson TE. 1996. ?*Brachychampsa sealeyi*, sp. nov., (Crocodylia, Alligatoroidea) from the Upper Cretaceous (lower Campanian) Menefee Formation, northwestern New Mexico. *Journal of Vertebrate Paleontology* 16(3):421–431.

Willis PMA. 1993. *Trilophosuchus rackhami* gen et sp. nov., a new crocodilian from the early Miocene limestones of Riversleigh, northwestern Queensland. *Journal of Vertebrate Paleontology* 13(1):90–98.

Willis PMA, Molnar RE. 1991. A new middle Tertiary crocodile from Lake Palankarinna, South Australia. *Records of the South Australian Museum* 25:39–55.

Wu XC, Brinkman DB, Russell AP. 1996. A new alligator from the Upper Cretaceous of Canada and the relationships of early eusuchians. *Palaeontology* 39(2):351–375.
