# Supplementary figures and images for: A new Mourasuchus (Alligatoroidea, Caimaninae) from the late Miocene of Venezuela, the phylogeny of Caimaninae and considerations on the feeding habits of Mourasuchus
Source: PeerJ. 2017 Mar 7;5:e3056. doi: 10.7717/peerj.3056 (PMC5344020; doi:10.7717/peerj.3056)

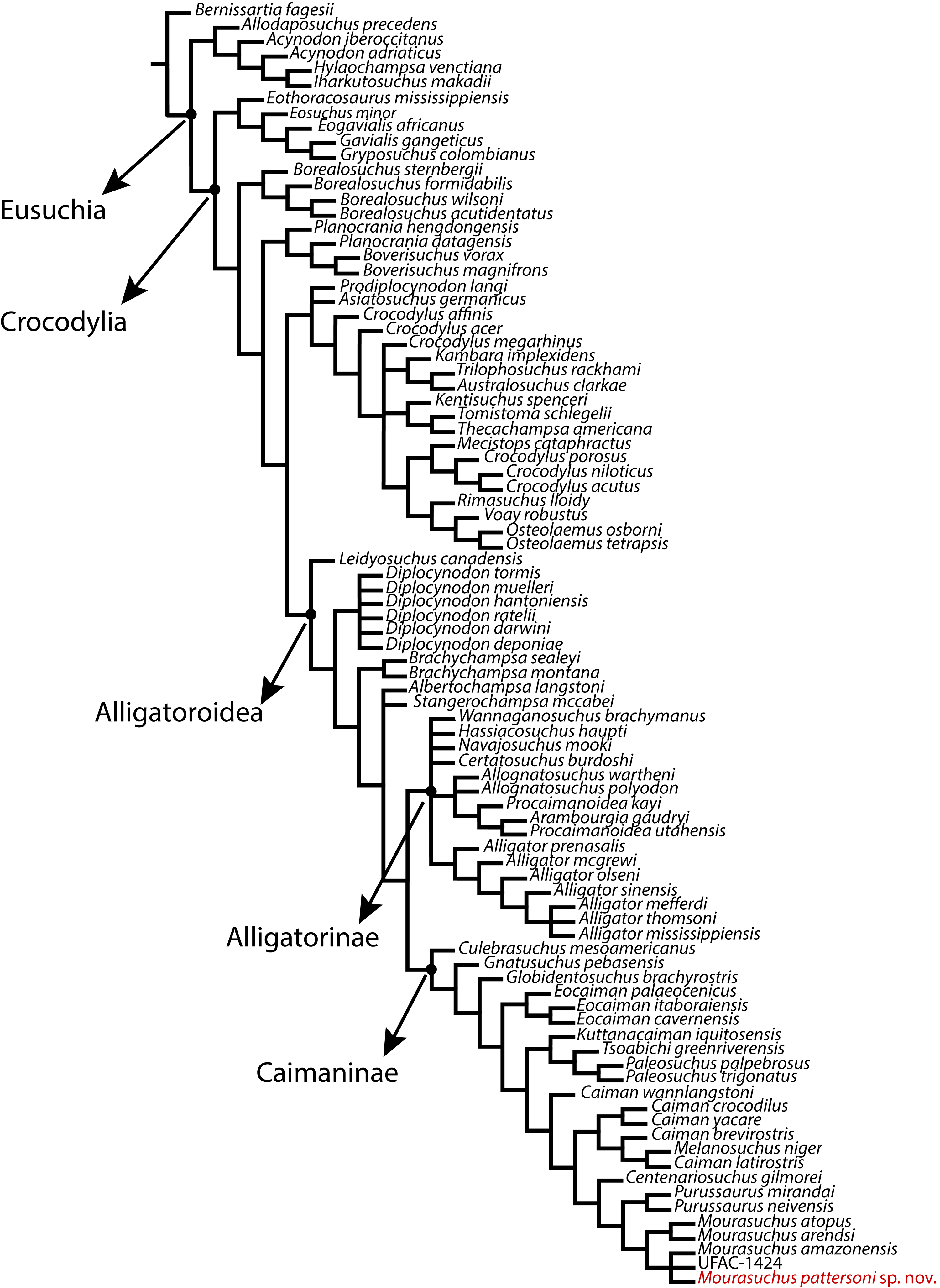

Supplement: Figure S1 [file peerj-05-3056-s003.png]
